# 6S-1 RNA Contributes to Sporulation and Parasporal Crystal Formation in *Bacillus thuringiensis*

**DOI:** 10.3389/fmicb.2020.604458

**Published:** 2020-11-26

**Authors:** Zhou Li, Li Zhu, Zhaoqing Yu, Lu Liu, Shan-Ho Chou, Jieping Wang, Jin He

**Affiliations:** ^1^State Key Laboratory of Agricultural Microbiology, College of Life Science and Technology, Huazhong Agricultural University, Wuhan, China; ^2^Agricultural BioResources Institute, Fujian Academy of Agricultural Sciences, Fuzhou, China

**Keywords:** non-coding RNA, 6S RNA, *Bacillus thuringiensis*, sporulation, parasporal crystal formation

## Abstract

6S RNA is a kind of high-abundance non-coding RNA that globally regulates bacterial transcription by interacting with RNA polymerase holoenzyme. Through bioinformatics analysis, we found that there are two tandem 6S RNA-encoding genes in the genomes of *Bacillus cereus* group bacteria. Using *Bacillus thuringiensis* BMB171 as the starting strain, we have explored the physiological functions of 6S RNAs, and found that the genes *ssrSA* and *ssrSB* encoding 6S-1 and 6S-2 RNAs were located in the same operon and are co-transcribed as a precursor that might be processed by specific ribonucleases to form mature 6S-1 and 6S-2 RNAs. We also constructed two single-gene deletion mutant strains Δ*ssrSA* and Δ*ssrSB* and a double-gene deletion mutant strain Δ*ssrSAB* by means of the markerless gene knockout method. Our data show that deletion of 6S-1 RNA inhibited the growth of *B. thuringiensis* in the stationary phase, leading to lysis of some bacterial cells. Furthermore, deletion of 6S-1 RNA also significantly reduced the spore number and parasporal crystal content. Our work reveals that *B. thuringiensis* 6S RNA played an important regulatory role in ensuring the sporulation and parasporal crystal formation.

## Introduction

In 1967, Hindley (1967) first discovered a highly abundant non-coding RNA in *Escherichia coli*. Because it exhibits a sedimentation coefficient of 6S, it was thus named 6S RNA, with its nucleotide sequence further determined by Brownlee (1971) afterwards. However, the study of 6S RNA remains silent for a long time until 2000, when Wassarman and Storz (2000) found that *E. coli* 6S RNA exists in high abundance throughout all bacterial growth phases, and exhibits a concentration as high as 10,000 molecules/cell in *E. coli* cells in the stationary phase. High abundant 6S RNA is found to regulate gene transcription in the stationary phase by combining with σ^70^-denpendent RNA polymerase holoenzyme, thus leading to the response regulation of bacterial to stresses such as starvation. Infact, all previous studies also seem to indicate that 6S RNA is a global regulatory factor (Wassarman and Storz, 2000; Cavanagh and Wassarman, 2014; Steuten et al., 2014; Wassarman, 2018). Later on, 6S RNA is found to exist in even more bacteria. Wehner et al. (2014) identified 1,750 6S RNA-encoding genes in 1,611 bacterial genomes, and found that the sizes of 6S RNA-encoding genes ranging from 153 to 237 base pairs (with an average of 183 bp). While most bacteria seem to contain only one 6S RNA-encoding gene in their genomes, some bacteria, such as *Bacillus* genus in *Firmicutes* phylum, do contain two 6S RNA-encoding genes. Interestingly, even four such 6S RNA-encoding genes are found in the genome of *Magnetococcus* sp. MC-1 of α-*Proteobacteria*. In fact, 6S RNA-encoding genes are now found to be widely distributed in various bacteria, even in bacteria from the phyla *Chloroflexi* and *Aquificae* at the root of the phylogenetic tree (Wehner et al., 2014), indicating that 6S RNAs are originated quite early and are widely distributed.

Besides, Trotochaud and Wassarman (2004) also reveal that the survival ability of *E. coli* is distinctively reduced in the stationary phase when the 6S RNA-encoding gene *ssrS1* is deleted, indicating that 6S RNA is beneficial to the normal growth and survival of *E. coli* in the stationary phase. Indeed, more and more regulatory functions of 6S RNA are gradually unveiled after many more in-depth researches. For example, it can now delay the sporulation of *Bacillus subtilis* (Cavanagh and Wassarman, 2013), promote the photosynthesis of *Synechocystis* (Heilmann et al., 2017), enhance the gene expression of pathogenicity island in *Salmonella enterica* serovar Typhimurium (Ren et al., 2017), increase the synthesis of antibiotics in *Streptomyces coelicolor* (Mikulík et al., 2014), optimize the symbiosis of *Bradyrhizobium* with leguminous plants (Madhugiri et al., 2012), and so on.

*B. subtilis* contains two kinds of 6S RNAs, called 6S-1 and 6S-2 RNAs, with their encoding genes *bsrA* and *bsrB* located in different regions of the genome. While deletion of 6S-1 RNA inhibits the growth of *B. subtilis* in the stationary phase (Hoch et al., 2015) and promotes earlier sporulation initiation by accelerating the utilization of nutrients (Cavanagh and Wassarman, 2013), the lack of 6S-2 RNA does not, however, seem to affect the growth and sporulation in the stationary phase (Cavanagh and Wassarman, 2013; Hoch et al., 2015). Until now, its physiological function remains unclear.

The *Bacillus cereus* group is a taxonomic group comprising closely related species of the *Bacillus genus* that includes more than 20 different species (Miller et al., 2016; Liu et al., 2017). Among them, the insect pathogen *Bacillus thuringiensis*, the anthracnose pathogen *Bacillus anthracis*, and the food-borne opportunistic pathogen *B. cereus* (Wang et al., 2019), have attracted extensive attention. Different from *B. subtilis* that harbors two 6S RNA-encoding genes located in different regions, *B. cereus* group bacteria contain the 6S RNA-encoding genes arranged in tandem in their genomes (Wehner et al., 2014). In this study, taking the *B. thuringiensis* BMB171 as a model to study the physiological function of 6S RNAs in *B. cereus* group bacteria, we found that the encoding genes *ssrSA* and *ssrSB* of 6S-1 and 6S-2 RNAs in BMB171 were located in the same operon and were co-transcribed as a precursor, which might be processed by ribonucleases to form mature 6S-1 and 6S-2 RNAs. Furthermore, deletion of the *ssrSA* gene inhibited the growth of *B. thuringiensis* in the stationary phase and decreased the sporulation and parasporal crystal formation. Besides, we found that diminished sporulation is primarily due to the decreased growth rate of the *ssrSA* deletion mutant in the stationary phase.

## Materials and Methods

### Bacterial Strains and Growth Conditions

The plasmids and strains used in this study are listed in Tables 1, 2, with primers used listed in Supplementary Table S1, respectively. *Escherichia coli* DH5α was used for cloning experiment and was cultured at 37°C in lysogeny broth (LB) medium (g/L: tryptone, 10; yeast extract, 5; and NaCl, 10). The medium was adjusted to pH 7.0 before autoclaving at 121°C for 15 min. *Bacillus thuringiensis* BMB171 and its derivative strains were cultured at 28°C in the GYS medium (g/L: glucose, 1.00; yeast extract, 2.00; K_2_HPO_4_·3H_2_O, 0.66; (NH_4_)_2_SO_4_, 2.00; MgSO_4_·7H_2_O, 0.04; MnSO_4_·H_2_O, 0.04; and CaCl_2_, 0.08), with the medium autoclaved at 115°C for 30 min after pH value being adjusted to 7.8. When necessary, relevant antibiotics were added to the cultures with the following final concentration: 50 μg/ml kanamycin, 25 μg/ml erythromycin, 100 μg/ml ampicillin, 300 μg/ml spectinomycin, or 60 U polymyxin (Wang et al., 2019). For the decoyinine-added experiment, cells were grown to an OD_600_ of 0.5 in GYS medium, followed by the addition of decoyinine to 0.4 mg/ml as previously described (Mitani et al., 1977; Ikehara et al., 1982; Cavanagh and Wassarman, 2013).

**Table 1:** Plasmids used in this study.

Plasmids	Relevant characteristics	Purposes	Origins
pHT1K	*B. thuringiensis-E. coli* shuttle plasmid; Amp^R^ Erm^R^	For β-galactosidase assays and gene complementation	Wang et al., 2013a
pHT1K-*lacZ*	pHT1K plasmid carrying the promoter-less *lacZ* gene, for β-galactosidase activity assay	β-galactosidase assays	Wang et al., 2013a
pHT1K-P1-*lacZ*	pHT1K carrying the promoter region (−190 to +10) of *ssrS* operon fused with *lacZ*	β-galactosidase assays	This work
pHT1K-P2-*lacZ*	pHT1K carrying the upstream region (+10 to +214) of *ssrSB* fused with *lacZ*	β-galactosidase assays	This work
pHT1K-P1-*ssrSA*	pHT1K carrying the promoter and *ssrSA* encoding region of *ssrS* operon fused with terminator region of *ssrS* operon	Gene complementation	This work
pHT1K-P1-*ssrSAB*	pHT1K carrying the promoter and encoding region of the *ssrS* operon	Gene complementation	This work
pRP1028	*B. thuringiensis-E. coli* shuttle plasmid; Amp^R^ Erm^R^; carrying *turbo-rfp* gene and an I*-Sce*I recognition site	Gene deletion	Janes and Stibitz, 2006
pSS4332	*B. thuringiensis-E. coli* shuttle plasmid; Km^R^; carrying *gfp* and I-*Sce*I restriction enzyme encoding gene	Gene deletion	Janes and Stibitz, 2006
pSS1827	The helper plasmid for conjugative transfer; Amp^R^	Gene deletion	Janes and Stibitz, 2006
pRP1028-*ssrSA*-UD	pRP1028 with the upstream and downstream regions of *ssrSA*, an intermediate plasmid in gene deletion experiment	Gene deletion	This work
pRP1028-*ssrSB*-UD	pRP1028 with the upstream and downstream regions of *ssrSB*, an intermediate plasmid in gene deletion experiments	Gene deletion	This work
pRP1028-*ssrSAB*-UD	pRP1028 with the upstream region of *ssrSA* and downstream region of *ssrSB*, an intermediate plasmid in gene deletion experiments	Gene deletion	This work
pBMB43-304	*B. thuringiensis*-*E. coli* shuttle plasmid; Amp^R^Erm^R^; carrying ORF of *cry1Ac10*;	Determination of the parasporal crystal protein Cry1Ac 10	Qi et al., 2015

**Table 2:** Strains used in this study.

Strains	Relevant characteristics	Origins
*E. coli* DH5α	F-Φ80*lacZ*ΔM15 Δ(*lacZYA*-*argF*) U169 *recA*1 *endA*1 *hsdR*17 (rk-, mk+) *phoA sup*E44 *thi*-1 *gyrA*96 *relA*1 λ-	Beijing TransGen Biotech Co., Ltd.
BMB171	*B. thuringiensis* strain BMB171; an acrystalliferous mutant strain; high transformation frequency	He et al., 2010; Wang et al., 2013a
Δ*ssrSA*	Markerless deletion of *ssrSA* in BMB171	This work
Δ*ssrSB*	Markerless deletion of *ssrSB* in BMB171	This work
Δ*ssrSAB*	Markerless deletion of *ssrSA* and *ssrSB* in BMB171	This work
BMB171/pHT1K	BMB171 strain harboring plasmid pHT1K	This work
Δ*ssrSA*/P1-*ssrSA*	Gene complemented strain: Δ*ssrSA* strain harboring plasmids pHT1K-P1-*ssrSA*	This work
Δ*ssrSAB/*P1-*ssrSAB*	Gene complemented strain: Δ*ssrSAB* strain harboring plasmid pHT1K-P1-*ssrSAB*	This work
BMB171/*lacZ*	BMB171 strain harboring pHT1K carrying the promoter-less *lacZ* gene	This work
BMB171/P1-*lacZ*	BMB171 strain harboring pHT1K carrying the promoter region of *ssrSA* fused with *lacZ*	This work
BMB171/P2-*lacZ*	BMB171 strain harboring pHT1K carrying the upstream region of *ssrSB* fused with *lacZ*	This work
BMB171-*cry*	BMB171 strain harboring plasmid pBMB43-304	This work
Δ*ssrSA*-*cry*	Δ*ssrSA* strain harboring plasmid pBMB43-304	This work
Δ*ssrSB*-*cry*	Δ*ssrSB* strain harboring plasmid pBMB43-304	This work
Δ*ssrSAB*-*cry*	Δ*ssrSAB* strain harboring plasmid pBMB43-304	This work

### RNA Extraction and RT-qPCR

The samples of 30 ml each from BMB171 and its derivative strains were cultured in GYS medium for 11 h before being centrifuged, followed by total RNA extraction and RT-qPCR experiments as previously described (Zheng et al., 2015, 2020; Fu et al., 2018). In these experiments, the *gapdh* gene was used as an internal control.

### Identification of Transcription Start Site

The 5'-rapid amplification of complementary DNA (cDNA) ends (5'-RACE) experiment was performed to identity the trancription start site (TSS) as described previously with some modifications (Ali et al., 2017). RNA was first extracted from BMB171 cells that were grown in GYS, followed by reverse transcription to cDNA. The 3'-end of cDNA was then labeled by poly(dA) using terminal deoxynucleotidyl transferase (Takara, Japan). The cDNA was then PCR amplified using primers of Primer-8 and *ssrSB*-R as listed in Supplementary Table S1. The PCR products were then cloned to the pMD19-T vector (Takara, Japan) and sequenced (Wang et al., 2019).

### Determination of β-Galactosidase Activity

BMB171/pHT1K, BMB171/P1-*lacZ*, and BMB171/P2-*lacZ* strains were grown at 28°C in a shaking incubator at 200 rpm in 100 ml GYS with 25 μg/ml erythromycin. Two milliliters of each culture was separately collected at indicated time and assayed for β-galactosidase activity as described previously (Zhou et al., 2017; Wang et al., 2019).

### Spore Count by Spread-Plate Method

BMB171 and its single deletion 6S-1 RNA mutant Δ*ssrSA*, single deletion 6S-2 RNA mutant Δ*ssrSB*, and the double deletion mutant Δ*ssrSAB* were cultured in GYS medium at 28°C for 24 h, which were then heated to 65°C for 30 min, followed by gradient dilution (10 times) with M9 minimum medium. Around 100 μl of each diluent was then spread onto LB plates. The colony-forming units (CFUs) per ml were then counted (Wang et al., 2016).

### Phase-Contrast Microscopic Analysis of Sporulation

BMB171 and its derivative strains were cultured at 28°C in GYS medium. To observe the morphology of vegetative cells and spores, 5 μl of each cell sample was collected at indicated time points, spotted onto the center of a glass slide, and covered with a coverslip. Spores were then observed with a phase-contrast microscope (Olympus, Japan; Zheng et al., 2020).

### Transmission Electron Microscope

To observe the morphology of BMB171 cells and its derivative strains, 4 ml of each sample was harvested by centrifugation at 17 h, with the cell pellets resuspended in 2.5% glutaraldehyde, and stored at 4°C overnight. Ultra-thin sections were finally prepared and stained as described (Craig et al., 1997). A Hitachi H-7000 FA transmission electron microscope (Hitachi, Japan) was then used for observation.

### Construction of Markerless Gene Deletion Strains

The markerless gene deletion mediated by homing endonuclease I-*Sce*I was performed in *B. thuringiensis* as previously reported (Zheng et al., 2015; Tang et al., 2016; Wang et al., 2016). Intermediate plasmids (pRP1028-*ssrSA*-UD, pRP1028-*ssrSB*-UD, and pRP1028-*ssrSAB*-UD) used in this study for gene deletion experiments were listed in Table 1. The helper plasmid pSS1827 for conjugational transfer and *B. thuringiensis-E. coli* shuttle plasmid pRP1028 carrying an I-*Sce*I recognition site and pSS4332 carrying I-*Sce*I restriction enzyme encoding gene was listed in Table 1.

### Observation of Parasporal Crystal and Determination of Parasporal Crystal Protein

Crystalliferous strains of BMB171-*cry*, Δ*ssrSA*-*cry*, Δ*ssrSB*-*cry*, and Δ*ssrSAB*-*cry* were obtained by transformation of the *cry1Ac10* gene with its original promoter in the plasmid pBMB43-304 (Qi et al., 2015) into BMB171, Δ*ssrSA*, Δ*ssrSB*, and Δ*ssrSAB* strains, respectively. To observe parasporal crystals, the crystalliferous strains were grown at 28°C and 200 rpm for 24 h in GYS medium supplemented with 25 mg/ml erythromycin. One drop from each culture was spotted onto the center of a glass slide, and covered with a coverslip. Parasporal crystals were then observed with a phase-contrast microscope (Olympus, Japan). To extract Cry1Ac10 protein, each culture was collected separately by centrifugation at 6,000 *g* for 15 min (AG Eppendorf, Hamburg, Germany). Procedure for the separation of Cry1Ac10 protein was carried out according to a previous study (Wang et al., 2013b). Finally, the Cry1Ac10 protein was visualized by SDS-PAGE, with its concentration measured by the Bradford method (Wang et al., 2016).

## Results

### The ssrSA and ssrSB Genes Were Located in the Same Operon and Were Co-Transcribed

**Figure 1:**
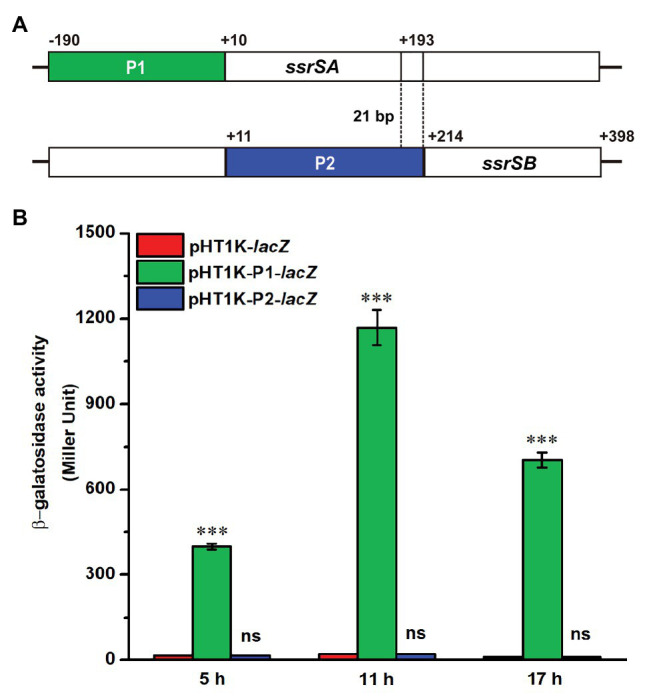
Promoter activity assays of the *ssrSA* and *ssrSB* genes in BMB171. **(A)** Schematic diagram of *ssrSA* and *ssrSB* genes and their putative promoter regions of P1 (marked in green) and P2 (marked in blue). The P1 and P2 regions were each fused with a β-galactosidase gene (*lacZ*) and transformed into the plasmid pHT1K, respectively. **(B)** The β-galactosidase activity assay of strains BMB171/P1-*lacZ* and BMB171/P2-*lacZ* harboring plasmids pHT1K-P1-*lacZ* and pHT1K-P2-*lacZ* with the BMB171/-*lacZ* harboring pHT1K carrying a promoter-less *lacZ* as the control. The strains were cultured at 28°C in GYS medium and were determined in the logarithmic phase (5 h), transition phase (11 h), and stationary phase (17 h). The values were means ± SDs for triplicate assays. Significances of differences by Student’s *t*-test are indicated. ^***^p < 0.001; ^**^p < 0.01; ^*^p < 0.05; ns, *p* > 0.05.

**Figure 2:**
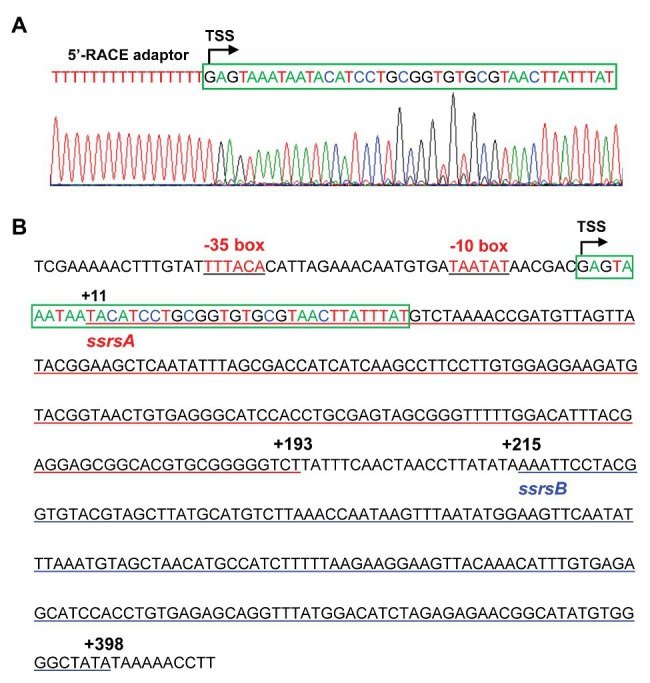
Identification of the transcription start site (TSS) and promoter regions of the *ssrSAB* operon. **(A)** TSS is identified by 5'-rapid amplification of complementary DNA (cDNA) ends (5'-RACE), with the 5'-RACE aptamer sequence (a tandem of 16 thymine nucleotides) shown in red, and the TSS marked with a curved arrow. The 5'-end 40 bp sequence of the *ssrSAB* operon was highlighted with a green box, with the colored nucleotides consistently as those in the sequencing diagram (below). **(B)** The promoter (−51 to −1) and encoding region (+1 to +398) sequences of the *ssrSAB* operon. The −35 and −10 regions were underlined in black, and the *ssrSA* (+1 to +193) and *ssrSB* (+215 to +398) encoding regions underlined in red and blue, respectively. The TSS was marked with a curved arrow with the 5'-end 40 bp sequence enclosed by a green box and colored as those in **(A)**.

The BMB171 strain was found to possess two 6S RNAs, with their encoding genes *ssrSA* (*BMB171_RS29145*) and *ssrSB* (*BMB171_RS291506*) located in tandem in the genome (Figure 1A). To explore whether the *ssrSA* and *ssrSB* genes are within the same operon, we first verified the co-transcription of the *ssrSA* and *ssrSB* genes by the semi-quantitative reverse transcription PCR (SqRT-PCR; Supplementary Figure S1), and found they were indeed co-transcribed. To further confirm that the *ssrSB* gene is not transcribed individually, we carried out the β-galactosidase assays to detect the promoter activities of the upstream regions of *ssrSA* and *ssrSB* genes in the BMB171 genome, respectively. We found that the upstream sequence of the *ssrSA* gene (P1 region) exhibited strong transcription initiation activities in the logarithmic phase (5 h), transition phase (11 h), and stationary phase (17 h), while those of the *ssrSB* gene (P2 region) basically exhibited no such transcription initiation activities at all when compared to the no promoter control (Figure 1B). These data indicate that the *ssrSB* gene was co-transcribed along, but not individually, with *ssrSA*. Finally, through the 5’-RACE experiment (Figure 2A), we identified a TSS as well as the canonical −35 and −10 regions (Figure 2B) upstream of the *ssrSA* gene, but found no independent TSS upstream of the *ssrSB* gene. These results thus clearly confirmed that the 6S RNA-encoding genes *ssrSA* and *ssrSB* were co-transcribed in BMB171.

### Deletion of 6S-1 RNA Inhibited the Growth of *B. thuringiensis* in the Stationary Phase

**Figure 3:**
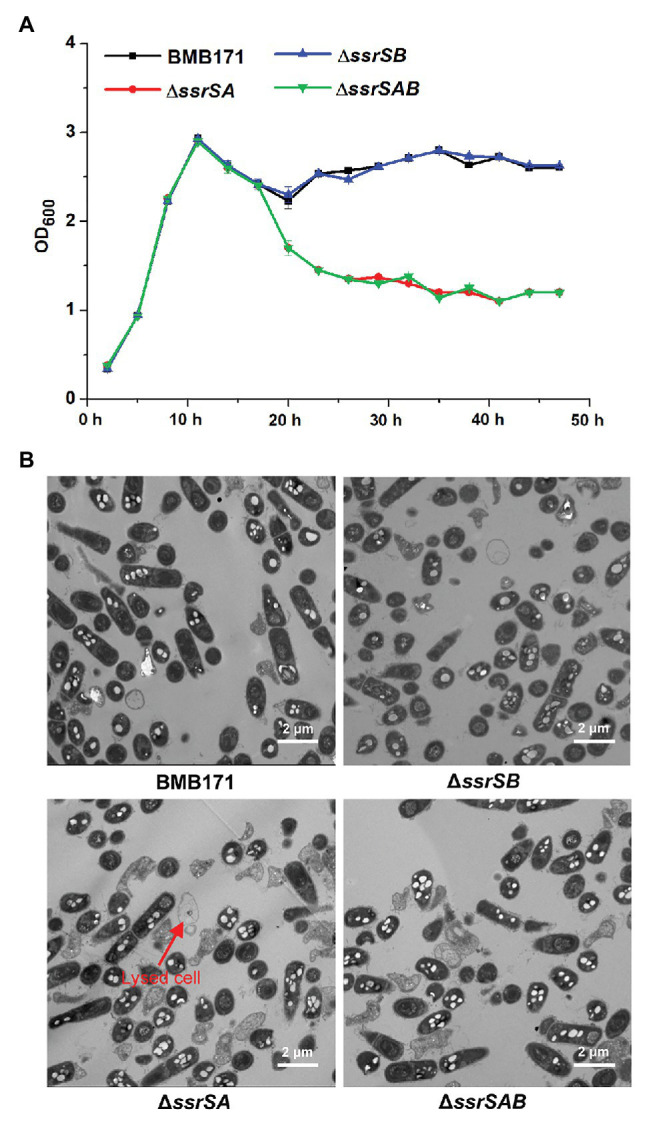
The effect of deleting of 6S RNA-encoding genes on the growths of *Bacillus thuringiensis*. **(A)** Determination of the growth curves of BMB171 and its 6S RNA-encoding genes deletion mutants Δ*ssrSA*, Δ*ssrSB*, and Δ*ssrSAB*. **(B)** Transmission electron microscope images of the cell morphologies of BMB171 and its deletion mutants Δ*ssrSA*, Δ*ssrSB*, and Δ*ssrSAB* at 17 h (stationary phase). Lysed cells are indicated by red arrows. The above-mentioned strains were cultured at 28°C in GYS medium. The values were means ± SDs for triplicate assays.

It has been reported that deletion of 6S-1 RNA results in inhibition of the growth of *B. subtilis* in the stationary phase (Hoch et al., 2015). We thus wonder whether deletion of 6S RNAs also exhibits the similar effect on the growth of *B. thuringiensis*. We have thus used the markerless gene knockout technology to precisely delete the two 6S RNA-encoding genes in the starting strain BMB171, and constructed the single deletion 6S-1 RNA mutant Δ*ssrSA*, single deletion 6S-2 RNA mutant Δ*ssrSB*, and the double deletion mutant Δ*ssrSAB*. We first determined the growth curves of BMB171 and its mutants, and found that the growth rates of mutants Δ*ssrSA* and Δ*ssrSAB* declined much more rapidly than that of starting strain BMB171 in the stationary phase, while Δ*ssrSB* exhibited no significant change compared to BMB171 (Figure 3A). Next, we checked the cell morphologies of BMB171, Δ*ssrSA*, Δ*ssrSB*, and Δ*ssrSAB* at 17 h with transmission electron microscope, and found that deletion of 6S-1 RNA caused more cells to lyse (Figure 3B), while the complemented strains Δ*ssrSA*/P1*-ssrSA* and Δ*ssrSAB*/P1-*ssrSAB* showed no significant differences in bacterial growth and morphology (Supplementary Figure S2) compared to the control strain BMB171/pHT1K. Taken together, these results indicate that deletion of 6S-1 RNA but not 6S-2 RNA inhibited the growth of *B. thuringiensis* in the stationary phase.

### Deletion of 6S-1 RNA Inhibited the Sporulation and Parasporal Crystal Formation

**Figure 4:**
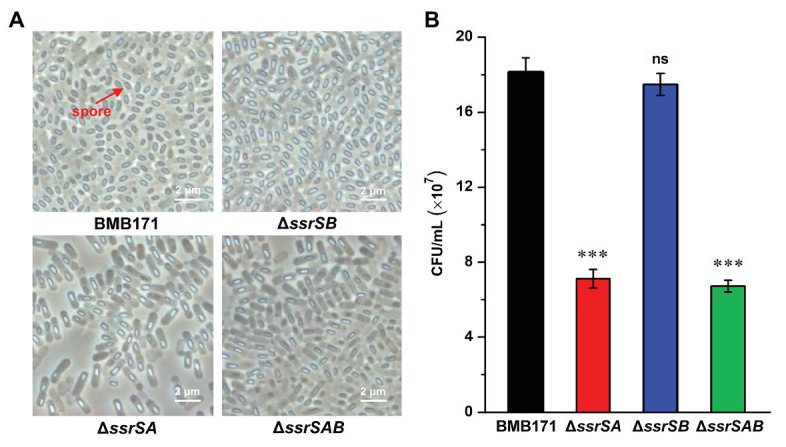
The effect of deleting of 6S RNA-encoding genes on the *B. thuringiensis* sporulation. **(A)** Observation of the spore formed by BMB171 and its deletion mutants Δ*ssrSA*, Δ*ssrSB*, and Δ*ssrSAB* at 24 h (stationary phase) using a phase contrast microscope. Oval-shaped spores are indicated by red arrows. **(B)** The spore counts of BMB171 and its mutants Δ*ssrSA*, Δ*ssrSB*, and Δ*ssrSAB* at 24 h (stationary phase). The strains were cultured at 28°C in GYS medium. The values were means ± SDs for triplicate assays. Significances of differences by Student’s *t*-test are indicated. ^***^p < 0.001; ^**^p < 0.01; ^*^p < 0.05; ns, *p* > 0.05.

To investigate the roles of 6S-1 and 6S-2 RNAs in the process of sporulation, we first checked the spores of BMB171 and its mutants in the stationary phase through a phase-contrast microscope, and found that the spores formed by mutants Δ*ssrSA* and Δ*ssrSAB* were much fewer than those of the starting strain BMB171. However, the spore amount of Δ*ssrSB* was similar to that of BMB171 (Figure 4A). Second, we counted the spores by spread-plate method, and found that the spore numbers formed by Δ*ssrSA* and Δ*ssrSAB* were much fewer than those of BMB171, whereas the spore number in Δ*ssrSB* did not differ much from that in BMB171 (Figure 4B). Meanwhile, we also complemented back the *ssrSA* and *ssrSAB* genes into the mutants Δ*ssrSA* and Δ*ssrSAB*, respectively. Phase-contrast microscopy observation and spore count did show that the sporulation capabilities of the complemented strains Δ*ssrSA*/P1-*ssrSA* and Δ*ssrSAB*/P1-*ssrSAB* have returned to the original level of the control strain BMB171/pHT1K (Supplementary Figure S3). These results indicate that deletion of the *ssrSA* gene, but not *ssrSB*, inhibited sporulation; namely, the presence of 6S-1 RNA was required for the normal sporulation.

**Figure 5:**
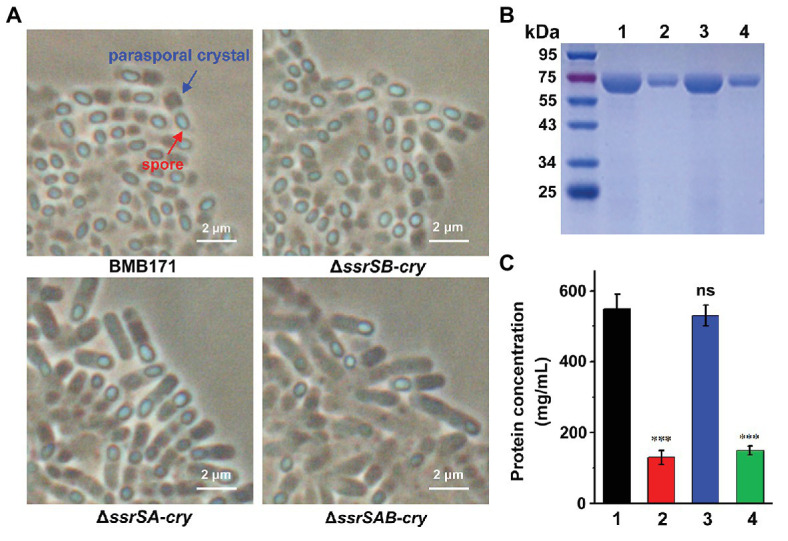
The effect of deleting 6S RNA-encoding genes on the parasporal crystal formation of *B. thuringiensis* **(A)** Phase contrast microscopy image of the parasporal crystal formation in crystalliferous strains BMB171-*cry*, Δ*ssrSA-cry*, Δ*ssrSB-cry*, and Δ*ssrSAB-cry* at 24 h (stationary phase). Cells with parasporal crystals and spores are indicated by blue and red arrows, respectively. **(B)** Separation of Cry1Ac10 from the four strains by SDS-PAGE. Lines 1–4 represent BMB171-*cry*, Δ*ssrSA-cry*, Δ*ssrSB-cry*, and Δ*ssrSAB-cry*, respectively. **(C)** The concentrations of Cry1Ac10 in BMB171-*cry* (1), Δ*ssrSA-cry* (2), Δ*ssrSB-cry* (3), and Δ*ssrSAB-cry* (4) at 24 h (stationary phase). The above-mentioned strains were cultured at 28°C in GYS medium. The values were means ± SDs for triplicate assays. Significances of differences by Student’s *t*-test are indicated. ^***^p < 0.001; ^**^p < 0.01; ^*^p < 0.05; ns, *p* > 0.05.

BMB171 is an acrystalliferous mutant of wild-type YBT-1463 (Li et al., 2000; Qi et al., 2015). To explore whether 6S RNAs affect the parasporal crystal formation, we introduced a pBMB43-304 plasmid containing the parasporal crystal protein encoding gene *cry1Ac10* and its original promoter sequence (Qi et al., 2015) into the BMB171 and its mutant strains Δ*ssrSA*, Δ*ssrSB*, and Δ*ssrSAB*, respectively, to obtain the crystalliferous strains of BMB171-*cry*, Δ*ssrSA*-*cry*, Δ*ssrSB-cry*, and Δ*ssrSAB-cry*. We first checked the parasporal crystal formation of BMB171 and its mutants in the stationary phase through a phase-contrast microscope, and found that the amounts of parasporal crystals formed by the Δ*ssrSA-cry* and Δ*ssrSAB-cry* strains were far less than that of BMB171-*cry*, while the amount of crystals in the Δ*ssrSB-cry* was similar to that of BMB171-*cry* (Figure 5A). Subsequently, we measured the content of parasporal crystal protein Cry1Ac10, and found that the concentrations of Cry1Ac10 in Δ*ssrSA-cry* and Δ*ssrSAB-cry* were remarkably reduced compared to BMB171-*cry*, while the Δ*ssrSB-cry* exhibited no substantial difference (Figures 5B,C). These experiments show that deletion of 6S-1 RNA not only inhibited the sporulation of bacterial cells, but also repressed the parasporal crystal formation.

### Reduction in Sporulation Capability Was Due to Inhibition of Bacterial Growth in the Stationary Phase

To figure out the reasons accounting for inhibition of sporulation in BMB171 after 6S-1 RNA deletion, we used the real-time quantitative PCR (RT-qPCR) to examine the transcription levels of several key early sporulation-related genes in BMB171 and its mutant’s strains Δ*ssrSA*, Δ*ssrSB*, and Δ*ssrSAB*. These relevant genes include *spo0A*, which encodes an essential transcriptional regulatory factor in the initial stage of sporulation (Lopez et al., 2009); *kinA*, which encodes a phosphorylation kinase that is mainly responsible for the phosphorylation of Spo0A (Jiang et al., 2000a); and *spo0H*, which encodes σ^H^ to promote the transcription of *spo0A* and *kinA* (Predich et al., 1992). Taking gene *gapdh* encoding glyceraldehyde-3-phosphate dehydrogenase as an internal control, we demonstrated that the transcription levels of *spo0A*, *kinA*, and *spo0H* from mutants Δ*ssrSA*, Δ*ssrSB*, and Δ*ssrSAB* at 0.5, 1.0, 1.5, and 2.0 h showed almost no difference to those of BMB171 (Supplementary Figure S4). Besides, we further determined the transcriptomes of BMB171 and Δ*ssrSAB* in the stationary phase and found that the transcription levels of sporulation-related genes did not change significantly between BMB171 and Δ*ssrSAB* (Supplementary Table S2). These data demonstrate that deletion of 6S-1 RNA did not change the start time of sporulation at the molecular level.

**Figure 6:**
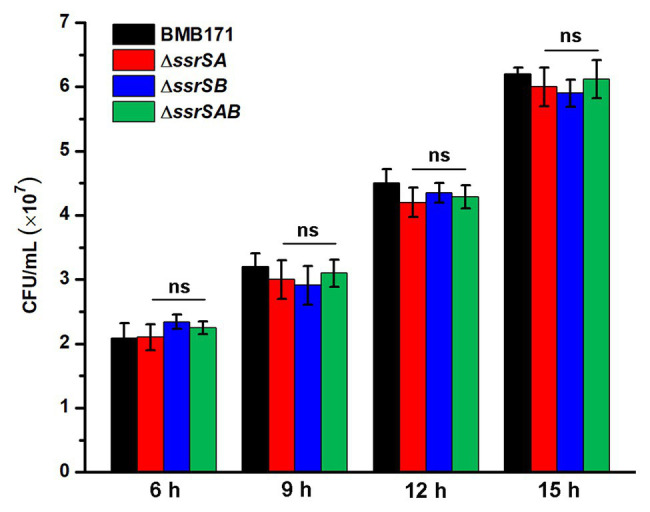
The effect of deleting 6S RNA-encoding genes on the sporulation of BMB171 and its mutants in the logarithmic phase. After adding the sporulation inducer decoyinine (0.4 mg/ml) to the GYS cultures of above-mentioned strains in the logarithmic phase (3 h, OD_600_ ≈ 0.5), the spore numbers were counted at 6, 9, 12, and 15 h after induction. The strains were cultured at 28°C and the numbers are revealed as means ± SDs in triplicate assays. Significances of differences by Student’s *t*-test are indicated. ^***^p < 0.001; ^**^p < 0.01; ^*^p < 0.05; ns, *p* > 0.05.

Since deletion of 6S-1 RNA did inhibit the growth of *B. thuringiensis* in the stationary phase, we wonder whether such inhibition also affects their sporulation? Because BMB171 and its various mutants Δ*ssrSA*, Δ*ssrSB*, and Δ*ssrSAB* exhibited no obvious difference in the growth curves in the logarithmic phase, we wonder whether there is difference in sporulation efficacy when these strains were induced to produce spores at this time? Given that decoyinine can induce *Bacillus* cells to produce spores in advance (Cavanagh and Wassarman, 2013; Wang et al., 2016; Zhou et al., 2017), we thus conducted an experiment by adding decoyinine to the GYS medium in the logarithmic phase. The results showed that the spore numbers formed by Δ*ssrSA*, Δ*ssrSB*, and Δ*ssrSAB* did not differ much from that of BMB171 (Figure 6). This experiment proves that the reduced sporulation efficacy was mainly caused by inhibition of the growth of *B. thuringiensis* in the stationary phase, not in the logarithmic phase. Further, we found that the transcription levels of genes involved in carbohydrate transport and metabolism, nucleotide transport and metabolism, protein translation, and energy production and conversion decreased significantly in Δ*ssrSAB* (Supplementary Table S3). Therefore, the decreased growth rate of the *ssrSA* deletion mutant in the stationary phase is the main reason for the diminished sporulation of *B. thuringiensis*.

## Discussion

In this study, we verified that the 6S-1 and 6S-2 RNAs-encoding genes *ssrSA* and *ssrSB* were located in the same operon and co-transcribed in *B. thuringiensis*. Through the phenotypic study of BMB171 and its deletion mutants of Δ*ssrSA*, Δ*ssrSB*, and Δ*ssrSAB*, we found that deletion of 6S-1 RNA not only inhibited the growth of *B. thuringiensis* in the stationary phase, but also decreased the sporulation and parasporal crystal formation. We further confirmed that inhibition of the growth in the stationary phase is likely the primary reason for the reduced sporulation efficacy.

In BMB171, we validated that the 6S-1 and 6S-2 RNAs-encoding genes *ssrSA* and *ssrSB* were arranged in tandem and co-transcribed, and verified that the upstream sequence of the *ssrSA* gene exhibited the transcription initiation activity, but not the upstream sequence of the *ssrSB* gene (Figure 1). These results indicate that the transcriptions of *ssrSA* and *ssrSB* genes were initiated through the upstream promoter region of the *ssrSA* gene, and were jointly transcribed into a long RNA precursor. In *E. coli*, the 6S RNA-encoding gene is first transcribed into a long RNA precursor, which is then cleaved by RNase E and RNase G into mature 6S RNA (Kim and Lee, 2004). Given the fact that RNase E and RNase G prefer to cut the regions enriched in nucleotides A and U on single-stranded RNA (Mackie, 1998; Jiang et al., 2000b; Tock et al., 2000), so we analyzed the RNA precursor sequence in BMB171 and found that, similar to *E. coli*, the 5'- and 3'-ends of the precursor RNA of 6S-1 RNA and 6S-2 RNA in BMB171 are also rich in A and U (Supplementary Figure S5). Moreover, transcriptomic data showed that 6S RNAs existed mainly as two separate mature 6S-1 and 6S-2 RNAs (Supplementary Figure S6). Combining the above results (Supplementary Figures S1, S5, S6), we suggest that a long precursor 6S RNA is first transcribed, followed by processing with RNase E, RNase G, or other ribonucleases to form mature and functional 6S-1 and 6S-2 RNAs.

In the present manuscript, we have analyzed the 6S RNA-encoding genes of *Bacillus genus* bacteria and their gene organization. *Bacillus* genus bacteria usually contain two different 6S RNA-encoding genes of *ssrSA* and *ssrSB* (Supplementary Tables S4–S6). However, unlike other *Bacillus* genus bacteria that have these two genes located in different regions of the genome, the two 6S RNA-encoding genes of *B. cereus* group bacteria were located in tandem positions. Furthermore, we also verified that they were in the same operon and are transcribed together. The *ssrSA* and *ssrSB* genes of *B. cereus* group bacteria are located between the genes encoding thiocyanase and glutathione spermidine synthase. The former is related to the detoxification of exogenous toxins while the latter involved in the resistance to stress such as oxidation (Supplementary Table S4). Other than the *B. cereus* group, there is also a *B. subtilis* group bacteria composed of more than 20 important bacteria species, such as, *B. subtilis*, *Bacillus licheniformis*, *Bacillus pumilus*, *Bacillus velezensis*, and *Bacillus amyloliquefaciens* in *Bacillus* genus (Caulier et al., 2019; Fayad et al., 2019). Unlike the *B. cereus* group bacteria, the *ssrSA* gene of *B. subtilis* group bacteria was mainly located between the FMN-dependent NADH-azoreductase and DNA helicase RecQ encoding genes. The former is related to the detoxification of exogenous toxins, while the latter is linked with DNA repair, recombination, and replication. The *ssrSB* gene, on the other hand, was mainly located between the aspartate-tRNA ligase and tRNA threonylcarbamoyladenosine dehydratase, both of which were associated with protein translation (Supplementary Table S5). Among *Bacillus* genus bacteria excluding *B. cereus* group and *B. subtilis* group, only the aspartate-tRNA ligase encoding gene locus upstream of the *ssrSB* gene was relatively conservative (Supplementary Table S6).

The 6S RNAs in *B. cereus* group bacteria were quite different to those of *B. subtilis* group bacteria regarding their gene organization, which might lead to their differences in sporulation. For example, in *B. subtilis*, deletion of 6S-1 RNA accelerates the utilization of nutrients, leading to the early arrival of nutrient deprivation conditions, which in turn induces the earlier expression of key early sporulation-related genes, ultimately promoting the earlier sporulation (Wassarman, 2018), while deletion of 6S-1 RNA in *B. thuringiensis* resulted in suppression of sporulation. In order to explore its possible regulatory mechanism, we have determined the transcriptomes of BMB171 and Δ*ssrSAB* in the stationary phase. After analyses, we found that the transcription levels of sporulation-related genes did not change significantly between BMB171 and Δ*ssrSAB* (Supplementary Table S2), indicating that 6S RNA does not affect sporulation by directly regulating the temporal expression of sporulation-related genes. Moreover, the experiments of decoyinine-induced sporulation in the logarithmic phase (Figure 6) and RT-qPCR detection of the transcription levels of key early sporulation-related genes (Supplementary Figure S4) further proved this conclusion. Since there is no available transcriptomic data from the 6S-1 RNA deletion in *B. subtilis*, we are currently unable to compare the 6S-1 RNA regulatory mechanisms between the two bacteria in a more comprehensive way. Altogether, the 6S RNAs of *B. subtilis* group and *B. cereus* group bacteria exhibited different gene organization and physiological functions, indicating that 6S RNAs might regulate the biological functions of *B. subtilis* group and *B. cereus* group bacteria *via* different mechanisms.

Then, how does deletion of 6S-1 RNA inhibit sporulation of *B. thuringiensis*? After further analyzing the transcriptomic data, we found that the transcription levels of genes involved in carbohydrate transport and metabolism, nucleotide transport and metabolism, protein translation, and energy production and conversion decreased significantly in Δ*ssrSAB* (Supplementary Table S3). According to our previous report that sporulation and parasporal crystal formation do require a lot of material and energy supply in *B. thuringiensis* (Wang et al., 2013c), we therefore speculate that the insufficient supply of material and energy in Δ*ssrSAB* was the main reason for inhibition of sporulation.

How does deletion of 6S-1 RNA inhibits the parasporal crystal formation of *B. thuringiensis*? In crystalliferous strain BMB171-*cry*, *cry1Ac10* with the original promoter was regulated by a sporulation-specific sigma factor SigE. In the transcriptomic data, we did not find a significant expression difference of *sigE* between BMB171 and Δ*ssrSAB* (Supplementary Table S2). Meanwhile, RT-qPCR assays confirmed that *cry1Ac10* had no expression difference between BMB171 and Δ*ssrSAB* (Supplementary Figure S7). However, the content of parasporal crystals was significantly reduced in Δ*ssrSAB* (Figure 5B). This indicates that deletion of 6S RNA may inhibit the translation of Cry1Ac10, further reducing the parasporal crystal formation.

Like 6S RNA, CsrA, CarD, and (p)ppGpp are all global regulatory factors that can respond to starvation stress in the stationary phase (Kalia et al., 2013; Romeo et al., 2013; Flentie et al., 2016). CsrA is believed to inhibit bacterial translation by interacting with conserved sequences on target mRNA under carbon starvation conditions (Romeo et al., 2013). Both CarD and (p)ppGpp, on the other hand, seem to regulate downstream gene transcription by interacting with RNA polymerase; in addition, the binding of CarD with RNA polymerase has been found to stabilize the transcription initiation complex to initiate the transcription of downstream genes (Flentie et al., 2016). Under starvation conditions, high levels of intracellular (p)ppGpp can also inhibit translation, which not only regulates the transcription of tRNA, rRNA, and ribosomal protein genes by binding to RNA polymerase, but also directly inhibits the bacterial translation activity through combining translation initiation factor IF2 with the translation elongation factors EF-Tu and EF-G (Kalia et al., 2013). Like CarD and (p)ppGpp, 6S RNA is also a global regulatory factor that can regulate gene transcription by binding to RNA polymerase. The consistent function of 6S RNA, CsrA, CarD, and (p)ppGpp may be to maintain and optimize the survival rates of bacteria under different stress conditions, which may be manifested in rather complex metabolic regulation networks. Currently we cannot figure out what are the specific roles 6S RNA plays in these networks and also how they co-regulate the bacterial response to starvation stress in the stationary phase, which are issues deserve further exploration.

## Data Availability Statement

The original contributions presented in the study are included in the article/Supplementary Material, further inquiries can be directed to the corresponding authors.

## Author Contributions

JH and ZL designed the experiments. ZL, LZ, ZY, and LL did the experiments. JH, S-HC, JW, and ZL wrote and revised the manuscript. All authors contributed to the article and approved the submitted version.

### Conflict of Interest

The authors declare that the research was conducted in the absence of any commercial or financial relationships that could be construed as a potential conflict of interest.
