# Supplementary material for: 6S-1 RNA Contributes to Sporulation and Parasporal Crystal Formation in Bacillus thuringiensis
Source: Front Microbiol. 2020 Nov 26;11:604458. doi: 10.3389/fmicb.2020.604458 (PMC7726162; doi:10.3389/fmicb.2020.604458)
Supplement: Supplementary file 1 [file Data_Sheet_1.pdf]

6S-1 RNA contributes to sporulation and parasporal crystal formation in *Bacillus thuringiensis*

Zhou Li[1], Li Zhu[1], Zhaoqing Yu[1], Lu Liu[1], Shan-Ho Chou[1], Jieping Wang [2,*], Jin He [1,*]

[1] State Key Laboratory of Agricultural Microbiology, College of Life Science and Technology, Huazhong Agricultural University, Wuhan, Hubei 430070, P. R. China

[2] Agricultural BioResources Institute, Fujian Academy of Agricultural Sciences, Fuzhou, Fujian 350000, P. R. China

* Correspondence: hejin@mail.hzau.edu.cn (J.H.); wangjpfaas@foxmail.com (J.P.W.)

Table S1. Primers used in this study.

Primers	Consequence*	Purposes
ssrSA-F	GCGGTGTGCGTAACTTATT	SqRT-PCR
ssrSA-R	AATGTCCAAAAACCCGCTAC	SqRT-PCR
ssrSB-F	ACGTAGCTTATGCATGTCTT	SqRT-PCR
ssrSB-R	ATGTCCATAAACCTGCTCTC	SqRT-PCR and 5'-RACE
ssrSAB-F	CAAGCCTTCCTTGTGGAGGA	SqRT-PCR
ssrSAB-R	AGCTACGTACACCGTAGGAA	SqRT-PCR
P1-F (*Nco* I)	CATGCCATGGTCATAAAGCAGGACATATTCCAACA	β-galactosidase assays
P1-R (*Bam*H I)	CGGGATCCTTATTTACTTGTCGTTATATTATCACA	β-galactosidase assays
P2-F (*Nco* I)	CATGCCATGGGAGTAAATAATACATCCTGCGGTG	β-galactosidase assays
P2-R (*Bam*H I)	CGGGATCCTATATAAGGTTAGTTGAAATAAGA	β-galactosidase assays
Primer-8	TTTTTTTTTTTTTTTT	5'-RACE
gapdh-F	TTTTGCTAGCGCTTTCGCAG	RT-qPCR, internal control
gapdh-R	TAGCGCCTGTTGTGAAGGTG	RT-qPCR, internal control
spo0A-F	CATGGAGATTGCTTCTCGTA	RT-qPCR
spo0A-R	TCGTCAAGTGAGTGGTAAAG	RT-qPCR
spo0H-F	ATGAGGAATCTGATCGAACG	RT-qPCR
spo0H-R	TCACATGCCTGTTTAACTGT	RT-qPCR
kinA-F	CGAGTCAATTTATGGCGATG	RT-qPCR
kinA-R	CATTTGGCATCGCTTCTATC	RT-qPCR
ssrSA-UF (*Hind* III)	CCCAAGCTTTGGTGGTTTCCCCGCTTGGAAAGAG	Plasmid pRP1028-*ssrSA*-UD construction
ssrSA-UR (*Mlu* I)	CGACGCGTTTATTTACTTGTCGTTATATTATCA	
ssrSA-DF (*Mlu* I)	CGACGCGTTATTTCAACTAACCTTATATAAAAT	
ssrSA-DR (*Bam*H I)	CGGGATCCTTATTAAAGAGAAAAATACTTATGTT	
ssrSB-UF (*Hind* III)	CCCAAGCTTCGATCATAAAGCAGGACATATTCCA	Plasmid pRP1028-*ssrSB*-UD construction
ssrSB-UR (*Mlu* I)	CGACGCGTTATATAAGGTTAGTTGAAATAAGA	
ssrSB-DF (*Mlu* I)	CGACGCGTTAAAAACCTTACGTTTTATACGTA	
ssrSB-DR (*Bam*H I)	CGGGATCCTGTCACCTTCGCACTCCCCTTTTTAA	
ssrSAB-UF (*Hind* III)	CCCAAGCTTTGGTGGTTTCCCCGCTTGGAAAGAG	Plasmid pRP1028-*ssrSAB*-UD construction
ssrSAB-UR (*Mlu* I)	CGACGCGTTTATTTACTTGTCGTTATATTATCA	
ssrSAB-DF (*Mlu* I)	CGACGCGTTAAAAACCTTACGTTTTATACGTA	
ssrSAB-DR (*Bam*H I)	CGGGATCCTGTCACCTTCGCACTCCCCTTTTTAA	
ssrSA-CF -1 (*Nco* I)	CATGCCATGGTCATAAAGCAGGACATATTCCAACA	Plasmid pHT1K-P1-*ssrSA* construction for gene complement
ssrSA-CR-1	AAAACGTAAGGTTTTTATATAGACCCCCGCACGTGCCGC TCCT	
ssrSA-CF -2	GAGCGGCACGTGCGGGGGTCTTAAAAACCTTACGTTTTA TAC	
ssrSA-CR-2 (*Kpn* I)	GGGGTACCGAGATTGATGAAGACAGAAAGTGCT	
ssrSAB-CF (*Nco* I)	CATGCCATGGTCATAAAGCAGGACATATTCCAACA	Plasmid pHT1K-P1-*ssrSAB* construction for gene complement
ssrSAB-CR (*Kpn* I)	GGGGTACCGAGATTGATGAAGACAGAAAGTGCT	

* Underlined sequences are the recognition sites for various restriction endonucleases.

Table S2. Comparing the transcription levels of sporulation-related genes of BMB171 with Δ*ssrSAB*.

GeneID	function	RPKM (Δ*ssrSAB*)	RPKM (BMB171)	log2 (Fold_change)	q-value	result
gi|296500839|ref|YP_003662539.1|	stage II sporulation protein R	34.44360409	36.80220113	0.075872205	0.449969299	ns
gi|296500840|ref|YP_003662540.1|	stage II sporulation protein D	33.50305476	34.72931917	0.119566667	0.254824457	ns
gi|296500844|ref|YP_003662544.1|	stage III sporulation protein D	76.40339355	143.057974	-0.733463141	1.70E-101	ns
gi|296500845|ref|YP_003662545.1|	stage V sporulation protein AE	3.601303104	2.672096882	0.601974864	0.092948152	ns
gi|296500846|ref|YP_003662546.1|	stage V sporulation protein AD	241.8980535	257.7200149	0.080022728	0.011401891	ns
gi|296500847|ref|YP_003662547.1|	stage V sporulation protein AC	58.58038917	56.64977328	0.219775746	0.00167899	ns
gi|296500848|ref|YP_003662548.1|	stage 0 sporulation regulatory protein	347.8469468	304.6102611	0.362916267	1.45E-28	ns
gi|296500849|ref|YP_003662549.1|	sporulation inhibitor KapD	376.4813233	313.8190642	0.43407345	1.04E-28	ns
gi|296500850|ref|YP_003662550.1|	stage VI sporulation protein D	143.4911682	135.8726854	0.250134634	4.55E-09	ns
gi|296500851|ref|YP_003662551.1|	sporulation initiation phosphotransferase B	137.3619476	104.9543644	0.55964839	1.06E-11	ns
gi|296500852|ref|YP_003662552.1|	stage V sporulation protein B	2.049674394	3.785821421	-0.713783464	0.127018245	ns
gi|296500853|ref|YP_003662553.1|	putative stage IV sporulation protein	1.929075379	2.503132061	-0.204396738	0.744539523	ns
gi|296500854|ref|YP_003662554.1|	stage III sporulation protein AA	4.811340946	4.422199975	0.293102922	0.512520239	ns
gi|296500862|ref|YP_003662562.1|	stage III sporulation protein SpoAB	11.62233203	14.14696885	-0.112165245	0.496389554	ns
gi|296500863|ref|YP_003662563.1|	stage III sporulation protein AD	14.04266975	18.81214256	-0.250418777	0.220341667	ns
gi|296500864|ref|YP_003662564.1|	stage III sporulation protein AE	90.79939164	116.4872188	-0.187988981	0.001401985	ns
gi|296500865|ref|YP_003662565.1|	stage III sporulation protein AF	145.0375242	185.2779436	-0.181836792	0.000305418	ns
gi|296500866|ref|YP_003662566.1|	stage III sporulation protein AG	112.4702349	139.1601391	-0.13577463	0.163011976	ns
gi|296500868|ref|YP_003662568.1|	stage IV sporulation protein B	7.41473776	9.417138177	-0.173464878	0.506570803	ns
gi|296500869|ref|YP_003662569.1|	stage 0 sporulation protein A	147.37897	94.96092577	0.805552923	7.56E-17	ns
gi|296500870|ref|YP_003662570.1|	stage II sporulation protein M	67.94836269	101.1918074	-0.403153649	2.38E-20	ns
gi|296500871|ref|YP_003662571.1|	sporulation sigma factor SigF	11.18260884	16.86591894	-0.421425974	0.025761309	ns
gi|296500872|ref|YP_003662572.1|	stage V sporulation protein AA	9.3122728	17.24787673	-0.717785395	0.001252839	ns
gi|296500873|ref|YP_003662573.1|	stage V sporulation protein AB	13.95781503	16.95885563	-0.109537552	0.577033827	ns
gi|296500874|ref|YP_003662574.1|	stage V sporulation protein AC	3.257678766	3.685166645	-0.00645736	0.988498414	ns
gi|296500875|ref|YP_003662575.1|	stage V sporulation protein AD	8.018901577	4.990570504	0.855628033	0.080655417	ns
gi|296500876|ref|YP_003662576.1|	stage V sporulation protein AE	13.61700268	18.65971173	-0.283089369	0.523186054	ns

gi|296500877|ref|YP_003662577.1|	stage V sporulation protein AE	12.91074115	23.56230102	-0.696480462	9.35E-06	ns
gi|296500878|ref|YP_003662578.1|	stage V sporulation protein D	319.3676314	279.9069332	0.361699028	4.75E-25	ns
gi|296500879|ref|YP_003662579.1|	sporulation sigma factor SigE	127.5967619	130.0609214	0.143832318	0.116587437	ns
gi|296500880|ref|YP_003662580.1|	sporulation sigma factor SigG	28231.42313	19524.84634	0.703418812	0	ns
gi|296500881|ref|YP_003662581.1|	stage V sporulation protein S	118.2439335	212.3704731	-0.673388876	1.11E-72	ns
gi|296500882|ref|YP_003662582.1|	stage 0 sporulation regulatory protein	87.20555465	139.9023265	-0.510499866	0.0017341	ns
gi|296500883|ref|YP_003662583.1|	stage V sporulation protein S	135.0121184	177.1177237	-0.220191528	5.53E-05	ns
gi|296500884|ref|YP_003662584.1|	stage II sporulation protein P	1473.820137	1293.725426	0.359457162	1.53E-81	ns
gi|296500892|ref|YP_003662592.1|	stage II sporulation protein P	99.20631906	101.2421929	0.142121412	0.065853132	ns
gi|296500893|ref|YP_003662593.1|	stage 0 sporulation regulatory protein	454.872192	451.8684338	0.180986613	7.26E-05	ns
gi|296500894|ref|YP_003662594.1|	stage IV sporulation protein A	1058.049514	995.9628158	0.258671508	3.53E-25	ns
gi|296500895|ref|YP_003662595.1|	stage 0 sporulation regulatory protein	916.7239425	1017.857339	0.02045204	0.1160103	ns
gi|296500896|ref|YP_003662596.1|	stage 0 sporulation regulatory protein	177.5758016	244.8518271	-0.292045813	1.97E-19	ns
gi|296500897|ref|YP_003662597.1|	stage II sporulation protein E	170.6564026	291.0522776	-0.598755614	2.14E-32	ns
gi|296500898|ref|YP_003662598.1|	stage V sporulation protein T	420.8405555	547.784645	-0.208906935	7.05E-30	ns
gi|296500899|ref|YP_003662599.1|	stage 0 sporulation protein J	12.16555791	16.6004465	-0.276991412	0.148191719	ns
gi|296500900|ref|YP_003662600.1|	sporulation initiation inhibitor protein soj	30.31803884	38.41201099	-0.16995291	0.147482401	ns
gi|296500901|ref|YP_003662601.1|	stage 0 sporulation protein J	92.79014682	86.77327459	0.268148994	2.61E-05	ns
gi|296500902|ref|YP_003662602.1|	sporulation initiation phosphotransferase F	24.10832856	34.93934822	-0.363892913	6.98E-05	ns
gi|296500903|ref|YP_003662603.1|	stage II sporulation protein Q	15.83323934	21.53668819	-0.272411817	0.164006047	ns
gi|296500904|ref|YP_003662604.1|	sporulation kinase	9.506945169	15.91494649	-0.57190041	0.001568499	ns
gi|296500905|ref|YP_003662605.1|	sporulation kinase	2.596047273	5.379523841	-0.879733675	0.010128656	ns
gi|296500906|ref|YP_003662606.1|	stage II sporulation protein B	4.572762057	5.183044298	-0.009305723	0.987683848	ns
gi|296500907|ref|YP_003662607.1|	stage IV sporulation protein FA	4.195936872	6.450989392	-0.449099321	0.263790635	ns
gi|296500908|ref|YP_003662608.1|	stage IV sporulation protein FB	4.952850974	7.094438462	-0.347001133	0.596567334	ns
gi|296500909|ref|YP_003662609.1|	sporulation sigma factor SigK	13.36483596	16.90537986	-0.167612152	0.593650751	ns
gi|296500910|ref|YP_003662610.1|	stage III sporulation protein AH	9.179532069	9.838865972	0.071356724	0.894938824	ns
gi|296500911|ref|YP_003662611.1|	stage V sporulation protein AF	17.73410926	15.61686005	0.354850608	0.120402464	ns
gi|296500926|ref|YP_003662626.1|	sporulation kinase B	31.13791217	63.90143805	-0.865748034	5.23E-19	ns
gi|296500927|ref|YP_003662627.1|	stage V sporulation protein E	79.20423839	121.5928669	-0.446980907	2.77E-08	ns

gi|296500928|ref|YP_003662628.1|	sporulation sigma-E factor processing peptidase	931.7599137	839.7428828	0.321438752	1.19E-44	ns
gi|296500929|ref|YP_003662629.1|	sporulation kinase	331.9825253	272.9703439	0.453791233	4.42E-09	ns
gi|296500930|ref|YP_003662630.1|	Transcriptional repressor of sporulation and protease synthase	231.5858776	223.039686	0.225674994	9.13E-05	ns
gi|296500931|ref|YP_003662631.1|	sporulation kinase	942.8446277	745.0736927	0.511065074	4.17E-65	ns
gi|296500932|ref|YP_003662632.1|	sporulation kinase B	150.1721568	135.9552389	0.314913756	4.14E-08	ns
gi|296500933|ref|YP_003662633.1|	sporulation kinase	196.1989931	191.0910365	0.209485681	0.000448006	ns
gi|296500934|ref|YP_003662634.1|	sporulation kinase	249.5883116	212.2655988	0.405107947	7.55E-11	ns
gi|296500935|ref|YP_003662635.1|	sporulation kinase	9.141547798	12.30175629	-0.256925789	0.333986947	ns
gi|296500936|ref|YP_003662636.1|	sporulation kinase	338.7543447	559.9860699	-0.553723339	0	ns
gi|296500937|ref|YP_003662637.1|	sporulation kinase D	648.6718597	881.2812806	-0.270685556	9.70E-08	ns
gi|296500939|ref|YP_003662639.1|	stage V sporulation protein R	2409.148551	2723.939564	-0.005743189	0.849960472	ns
gi|296500940|ref|YP_003662640.1|	sporulation kinase	690.7492034	889.8685206	-0.194002047	4.54E-11	ns
gi|296500941|ref|YP_003662641.1|	Jag protein	512.202999	718.0288333	-0.315897927	4.55E-92	ns
gi|296500942|ref|YP_003662642.1|	export protein for polysaccharides and teichoic acids	2263.760215	2134.777445	0.256063628	3.61E-125	ns
gi|296500943|ref|YP_003662643.1|	export protein for polysaccharides and teichoic acids	386.0750769	433.2428925	0.005133493	0.942672931	ns
gi|296500944|ref|YP_003662644.1|	polysaccharides export protein	285.6021107	361.5877256	-0.168910913	3.38E-05	ns
gi|296500945|ref|YP_003662645.1|	polysaccharides export protein	187.0177478	264.6361978	-0.329407073	6.56E-12	ns
gi|296500946|ref|YP_003662646.1|	Transcriptional regulator, AbrB	211.30219	358.086296	-0.589571437	3.84E-21	ns
gi|296500947|ref|YP_003662647.1|	chitooligosaccharide deacetylase	157.7147141	256.2757045	-0.5289513	8.07E-35	ns
gi|296500975|ref|YP_003662675.1|	cell division protein ftsH	7.226519999	14.68240308	-0.851286988	4.27E-05	ns
gi|296500976|ref|YP_003662676.1|	peptidoglycan N-acetylglucosamine deacetylase	1116.165191	895.6194046	0.489021019	1.02E-76	ns
gi|296500977|ref|YP_003662677.1|	transcription state transcriptional regulator AbrB	905.1564704	694.3559793	0.55391987	4.07E-70	ns
gi|296500978|ref|YP_003662678.1|	sporulation-specific protease YabG	0.504686113	0.337357913	0.75253454	0.662839295	ns
gi|296500979|ref|YP_003662679.1|	transcription state transcriptional regulator AbrB	4.043143652	2.635077143	0.789060416	0.053124066	ns

“ns” indicates not significant

Table S3. Comparing the differential expression genes related to carbohydrate transport and metabolism, nucleotide transport and metabolism, protein translation, and energy production and conversion of BMB171 with Δ*ssrSAB*.

GeneID	function	RPKM (Δ*ssrSAB*)	RPKM (BMB171)	log2 (Fold_change)	q-value	result
**Carbohydrate transport and metabolism**						
gi|296505558|ref|YP_003667258.1|	glyceraldehyde-3-phosphate dehydrogenase	69.65672714	166.4715279	-1.085512706	3.68E-93	down
gi|296505330|ref|YP_003667030.1|	glycogen phosphorylase	308.7227176	704.0174401	-1.017871368	0	down
gi|296505557|ref|YP_003667257.1|	phosphoglycerate kinase	25.82289318	107.8426489	-1.890777134	1.59E-162	down
gi|296505556|ref|YP_003667256.1|	triosephosphate isomerase	25.39318833	76.57489133	-1.421001715	1.17E-49	down
gi|296505341|ref|YP_003667041.1|	glucose-6-phosphate isomerase	56.13231104	129.9971391	-1.040148359	1.54E-72	down
gi|296505070|ref|YP_003666770.1|	6-phosphofructokinase	78.03393598	177.440774	-1.013735837	6.81E-85	down
gi|296505331|ref|YP_003667031.1|	glycogen synthase	117.8248224	360.8012455	-1.443132662	0	down
gi|296505069|ref|YP_003666769.1|	pyruvate kinase	66.54593579	170.1236658	-1.182733233	4.38E-190	down
gi|296505311|ref|YP_003667011.1|	2-dehydro-3-deoxygluconokinase	8.515647693	24.97493469	-1.380864585	1.80E-21	down
gi|296503627|ref|YP_003665327.1|	phosphoenolpyruvate synthase	23.08779257	118.6902004	-2.190570525	0	down
gi|296503022|ref|YP_003664722.1|	phosphoglyceromutase	136.5169098	331.9101311	-1.110284853	5.93E-141	down
gi|296505554|ref|YP_003667254.1|	phosphoglyceromutase	37.59855839	104.4182318	-1.302196231	2.42E-105	down
gi|296502596|ref|YP_003664296.1|	peptidoglycan N-acetylglucosamine deacetylase	12.02835237	34.33293942	-1.341726191	9.10E-23	down
gi|296504582|ref|YP_003666282.1|	phosphopentomutase	107.1833379	664.5639134	-2.460899154	0	down
gi|296501498|ref|YP_003663198.1|	PTS system sucrose-specific transporter subunit IIBC	2.253678221	5.097485413	-1.006076048	0.000206011	down
gi|296505170|ref|YP_003666870.1|	pullulanase	5.727786841	17.9387944	-1.475605075	3.38E-35	down
gi|296504548|ref|YP_003666248.1|	N-acetylglucosamine-6-phosphate deacetylase	26.94602175	67.26194502	-1.148290055	1.24E-47	down
gi|296501418|ref|YP_003663118.1|	D-ribose pyranase	2.369220921	9.502247879	-1.83242796	1.46E-05	down
gi|296504260|ref|YP_003665960.1|	6-phospho-beta-glucosidase	5.27200125	15.75569121	-1.408022279	1.92E-19	down
gi|296502912|ref|YP_003664612.1|	multidrug resistance protein B	39.59017445	177.3431139	-1.991900832	0	down
gi|296501325|ref|YP_003663025.1|	SN-glycerol-3-phosphate transport ATP-binding protein UgpC	3.417892476	7.842649731	-1.026806006	2.48E-05	down
**Nucleotide transport and metabolism**						
gi|296504581|ref|YP_003666281.1|	purine nucleoside phosphorylase	76.03323466	623.0988338	-2.863330792	0	down

gi|296504383|ref|YP_003666083.1|	dihydroorotase	3.308497854	10.00828475	-1.425518342	6.74E-12	down
gi|296501110|ref|YP_003662810.1|	phosphoribosylformylglycinamidine synthase II	3.380942287	10.03958913	-1.398774783	2.55E-19	down
gi|296501112|ref|YP_003662812.1|	phosphoribosylaminoimidazole	1.178570549	4.379333483	-1.722245043	4.44E-06	down
gi|296504379|ref|YP_003666079.1|	dihydroorotate dehydrogenase 1B	21.03021607	103.1760205	-2.123143979	4.20E-142	down
gi|296502032|ref|YP_003663732.1|	ribonucleotide-diphosphate reductase subunit alpha	66.1823546	176.8997711	-1.246985505	3.63E-250	down
gi|296502518|ref|YP_003664218.1|	pyrimidine-nucleoside phosphorylase	18.45825501	93.42754282	-2.168153603	1.21E-184	down
gi|296504580|ref|YP_003666280.1|	pyrimidine-nucleoside phosphorylase	133.39028	543.0295216	-1.853948938	0	down
gi|296504378|ref|YP_003666078.1|	orotidine 5'-phosphate decarboxylase	80.423879	230.916551	-1.350247604	9.89E-130	down
gi|296504320|ref|YP_003666020.1|	uridylate kinase	42.12418837	103.1902265	-1.121157389	2.49E-44	down
gi|296504384|ref|YP_003666084.1|	aspartate carbamoyltransferase	3.549349879	9.253008591	-1.210939584	1.44E-06	down
gi|296500964|ref|YP_003662664.1|	aspartate carbamoyltransferase	97.88972479	250.2146328	-1.182508674	5.92E-104	down
gi|296502031|ref|YP_003663731.1|	ribonucleotide reductase stimulatory protein	92.81878576	282.4177711	-1.433913992	1.33E-87	down
gi|296502517|ref|YP_003664217.1|	nucleoside permease NupC	33.37346321	122.6968779	-1.706897078	6.26E-71	down
gi|296504243|ref|YP_003665943.1|	ADA regulatory protein	1.813319452	7.151479173	-1.808180414	4.34E-06	down
**Protein translation**						
gi|296505036|ref|YP_003666736.1|	phenylalanyl-tRNA synthetase subunit alpha	3.974585999	11.74592246	-1.391855369	6.39E-11	down
gi|296500965|ref|YP_003662665.1|	methionine aminopeptidase	161.6340041	386.7124271	-1.087102236	1.70E-160	down
gi|296500948|ref|YP_003662648.1|	50S ribosomal protein L22	81.2441344	205.8756256	-1.17000932	8.83E-45	down
gi|296500949|ref|YP_003662649.1|	30S ribosomal protein S3	46.03578497	145.9325992	-1.493046393	3.86E-88	down
gi|296500953|ref|YP_003662653.1|	50S ribosomal protein L14	27.38161514	123.3509439	-2.00006115	5.30E-63	down
gi|296500955|ref|YP_003662655.1|	50S ribosomal protein L5	20.71549574	97.82442368	-2.068056209	3.97E-76	down
gi|296500957|ref|YP_003662657.1|	30S ribosomal protein S8	21.88617498	92.13168505	-1.90224941	7.17E-48	down
gi|296500960|ref|YP_003662660.1|	30S ribosomal protein S5	220.1116457	523.2976758	-1.077968223	8.69E-144	down
gi|296504360|ref|YP_003666060.1|	sun protein	5.892541159	18.86323503	-1.507187132	3.81E-24	down
gi|304570848|ref|YP_003858733.1|	30S ribosomal protein S19	90.7946598	219.424116	-1.101614593	1.44E-35	down
gi|296500952|ref|YP_003662652.1|	30S ribosomal protein S17	58.42342578	196.4145963	-1.577855133	1.69E-41	down
gi|296500950|ref|YP_003662650.1|	50S ribosomal protein L16	33.34757001	110.9570175	-1.56291922	9.93E-48	down
gi|296500954|ref|YP_003662654.1|	50S ribosomal protein L24	22.66881792	107.1533071	-2.069467158	1.32E-48	down
gi|296500956|ref|YP_003662656.1|	30S ribosomal protein S14	27.54880703	177.795782	-2.518731196	1.10E-60	down
gi|296500962|ref|YP_003662662.1|	50S ribosomal protein L15	210.6189047	477.3350548	-1.008939203	7.20E-104	down

gi|296500924|ref|YP_003662624.1|	cysteinyl-tRNA synthetase	20.1139194	53.226825	-1.232531128	2.16E-46	down
gi|296500951|ref|YP_003662651.1|	50S ribosomal protein L29P	62.11656595	207.0093611	-1.565217879	2.22E-41	down
gi|296500959|ref|YP_003662659.1|	50S ribosomal protein L18	157.4620673	398.8950631	-1.169576792	6.20E-91	down
gi|296500967|ref|YP_003662667.1|	50S ribosomal protein L36	310.837948	735.0585029	-1.070268284	3.91E-46	down
gi|296505897|ref|YP_003667597.1|	50S ribosomal protein L9	47.1446831	174.999323	-1.720754209	7.16E-88	down
gi|296500966|ref|YP_003662666.1|	translation initiation factor IF-1	122.9198749	320.182698	-1.209748996	5.68E-47	down
gi|296503566|ref|YP_003665266.1|	tetracycline resistance protein	11.99122782	40.20181795	-1.573853203	2.13E-77	down
gi|296504308|ref|YP_003666008.1|	translation initiation factor IF-2	156.6312085	358.4457288	-1.022954854	0	down
gi|296505915|ref|YP_003667615.1|	ribonuclease P	14.10220622	33.6863509	-1.084815122	1.82E-07	down
gi|296504271|ref|YP_003665971.1|	tRNA 2-methylthioadenosine synthase	34.19825673	82.01170862	-1.090478959	1.22E-70	down
gi|296504665|ref|YP_003666365.1|	hemolysin A	86.08863622	202.3587222	-1.061592165	2.67E-91	down
gi|296500912|ref|YP_003662612.1|	lysyl-tRNA synthetase	33.00579889	80.13026354	-1.108199552	1.77E-69	down
gi|296504309|ref|YP_003666009.1|	hypothetical protein	174.4111093	483.5182286	-1.299650192	8.24E-48	down
gi|296503308|ref|YP_003665008.1|	acetyltransferase	7.736829663	18.42414973	-1.080355474	5.04E-07	down
gi|296500961|ref|YP_003662661.1|	50S ribosomal protein L30	206.6510374	493.0983638	-1.083250709	1.51E-50	down
gi|296501251|ref|YP_003662951.1|	tRNA(Uracil-5-)-methyltransferase	16.94428768	39.79979977	-1.060534033	3.60E-30	down
**Energy production and conversion**						
gi|296503317|ref|YP_003665017.1|	acetoin dehydrogenase E1 component subunit beta	215.8827764	859.1303286	-1.821200705	0	down
gi|296503316|ref|YP_003665016.1|	branched-chain alpha-keto acid dehydrogenase subunit E2	89.9720757	704.7579696	-2.798150524	0	down
gi|296504464|ref|YP_003666164.1|	branched-chain alpha-keto acid dehydrogenase subunit E2	6.825390645	22.04555505	-1.520076125	1.83E-27	down
gi|296505065|ref|YP_003666765.1|	isocitrate dehydrogenase	263.8423278	1037.934453	-1.804539251	0	down
gi|296501946|ref|YP_003663646.1|	alpha-ketoglutarate decarboxylase	64.34413598	153.3051752	-1.08109767	1.21E-241	down
gi|296505417|ref|YP_003667117.1|	IscU protein	56.63349239	165.1624687	-1.372730334	6.54E-58	down
gi|296504120|ref|YP_003665820.1|	aconitate hydratase	349.9349737	789.4344212	-1.002304415	0	down
gi|296503318|ref|YP_003665018.1|	acetoin dehydrogenase E1 component subunit alpha	205.0238079	802.1530306	-1.796657893	0	down
gi|296504652|ref|YP_003666352.1|	2-oxoisovalerate dehydrogenase subunit alpha	361.4267795	944.4779194	-1.214385513	0	down
gi|296501984|ref|YP_003663684.1|	iron-sulfur cluster-binding protein	7.206860911	20.0500206	-1.30473266	4.62E-22	down
gi|296502738|ref|YP_003664438.1|	respiratory nitrate reductase subunit beta	3.269843362	14.63658421	-1.990857323	1.57E-25	down
gi|296503315|ref|YP_003665015.1|	dihydrolipoamide dehydrogenase	825.7202413	1881.392643	-1.016647841	0	down
gi|296504653|ref|YP_003666353.1|	dihydrolipoamide dehydrogenase	155.5565401	556.2099623	-1.666762382	0	down

gi|296505261|ref|YP_003666961.1|	cytochrome d ubiquinol oxidase subunit I	29.29572043	84.85263709	-1.362841418	2.43E-91	down
gi|296502562|ref|YP_003664262.1|	cytochrome d ubiquinol oxidase subunit II	2.909512962	8.965361171	-1.452155879	6.21E-09	down
gi|296502773|ref|YP_003664473.1|	HEAT repeat-containing PBS lyase	19.19417931	56.96683139	-1.398025136	1.09E-53	down
gi|296504861|ref|YP_003666561.1|	bifunctional acetaldehyde-CoA/alcohol dehydrogenase	1.773766248	7.808786298	-1.966854281	3.90E-28	down
gi|296501272|ref|YP_003662972.1|	formate acetyltransferase	148.9270401	1212.679798	-2.854092886	0	down
gi|296502739|ref|YP_003664439.1|	respiratory nitrate reductase subunit delta	4.892889098	22.21320788	-2.011231113	3.21E-17	down
gi|296502740|ref|YP_003664440.1|	respiratory nitrate reductase subunit gamma	8.367549472	22.33822754	-1.245209486	1.36E-11	down
gi|296504654|ref|YP_003666354.1|	butyrate kinase	147.281945	536.4320839	-1.693386792	0	down
gi|296502564|ref|YP_003664264.1|	transport ATP-binding protein CydC	1.882698631	4.3627712	-1.041014582	7.15E-05	down
gi|296502563|ref|YP_003664263.1|	transport ATP-binding protein CydD	1.131587156	4.412394653	-1.791785976	6.00E-10	down
gi|296502736|ref|YP_003664436.1|	respiratory nitrate reductase subunit alpha	4.005936809	15.13646012	-1.746388129	9.24E-60	down

Table S4. Gene organization of *ssrSA* and *ssrSB* in *B. cereus* group bacteria.

Strains	Expression product of the upstream gene for *ssrSA*	The Location of *ssrSA* on the genome	The Location of *ssrSB* on the genome	Expression product of the downstream gene for *ssrSB*
Bacillus thuringiensis BMB171	sulfurtransferase	1,533,736..1,533,918	1,533,940..1,534,123	glutathionylspermidine synthase
Bacillus thuringiensis ATCC 10792	sulfurtransferase	1,392,463..1,392,645	1,392,667..1,392,850	glutathionylspermidine synthase
Bacillus thuringiensis MYBT 18246	sulfurtransferase	1,671,978..1,672,160	1,672,182..1,672,365	glutathionylspermidine synthase
Bacillus thuringiensis HD12	sulfurtransferase	1,624,669..1,624,851	1,624,873..1,625,056	glutathionylspermidine synthase
Bacillus thuringiensis L-7601	sulfurtransferase	1,597,066..1,597,248	1,597,270..1,597,453	glutathionylspermidine synthase
Bacillus thuringiensis YC-10	sulfurtransferase	1,047,976..1,048,158 (complement)	1,047,771..1,047,954 (complement)	glutathionylspermidine synthase
Bacillus thuringiensis YWC2-8	sulfurtransferase	4,232,290..4,233,123 (complement)	4,231,786..4,231,969 (complement)	glutathionylspermidine synthase
Bacillus thuringiensis C15	sulfurtransferase	1,641,831..1,642,013	1,642,035..1,642,218	glutathionylspermidine synthase
Bacillus thuringiensis Bc601	sulfurtransferase	1,690,827..1,691,009	1,691,031..1,691,214	glutathionylspermidine synthase
Bacillus albus PG 26	sulfurtransferase	1,023,128..1,023,310 (complement)	1,022,923..1,023,106 (complement)	glutathionylspermidine synthase
Bacillus albus DLOU-Yingkou	sulfurtransferase	3,114,632..3,114,814	3,114,836..3,115,019	glutathionylspermidine synthase
Bacillus anthracis MCCC 1A01412	sulfurtransferase	1,512,208..1,512,391	1,512,413..1,512,596	glutathionylspermidine synthase
Bacillus anthracis 14RA5914	sulfurtransferase	4,500,344..4,500,526	4,500,548..4,500,731	glutathionylspermidine synthase
Bacillus anthracis MCCC 1A02161	sulfurtransferase	1,543,637..1,543,819	1,543,841..1,544,024	glutathionylspermidine synthase
Bacillus anthracis FDAARGOS_341	sulfurtransferase	4,307,550..4,307,732	4,307,754..4,307,937	glutathionylspermidine synthase
Bacillus anthracis SPV842_15	sulfurtransferase	1,507,263..1,507,445	1,507,467..1,507,650	glutathionylspermidine synthase
Bacillus anthracis Parent1	sulfurtransferase	1,507,260..1,507,442	1,507,464..1,507,647	glutathionylspermidine synthase
Bacillus anthracis PR05	sulfurtransferase	1,507,260..1,507,442	1,507,464..1,507,647	glutathionylspermidine synthase
Bacillus anthracis PR02	sulfurtransferase	1,507,260..1,507,442	1,507,464..1,507,647	glutathionylspermidine synthase

Bacillus anthracis London_499	sulfurtransferase	1,507,407..1,507,589	1,507,611..1,507,794	glutathionylspermidine synthase
Bacillus anthracis Shikan-NIID	sulfurtransferase	3,962,438..3,962,620 (complement)	3,962,233..3,962,416 (complement)	glutathionylspermidine synthase
Bacillus cereus G1-1	sulfurtransferase	3,846,105..3,846,287 (complement)	3,845,900..3,846,083 (complement)	glutathionylspermidine synthase
Bacillus cereus DLOU-Tangshan	sulfurtransferase	2,210,249..2,210,431	2,210,453..2,210,636	glutathionylspermidine synthase
Bacillus cereus CMCC P0021	sulfurtransferase	1,594,338..1,594,520	1,594,542..1,594,725	glutathionylspermidine synthase
Bacillus cereus M13	sulfurtransferase	1,514,386..1,514,568	1,514,590..1,514,773	glutathionylspermidine synthase
Bacillus cereus HBL-AI	sulfurtransferase	1,534,311..1,534,493	1,534,515..1,534,698	glutathionylspermidine synthase
Bacillus cereus ZB201708	sulfurtransferase	1,473,657..1,473,839	1,473,861..1,474,044	glutathionylspermidine synthase
Bacillus cereus SB1	sulfurtransferase	1,503,418..1,503,600	1,503,622..1,503,805	glutathionylspermidine synthase
Bacillus cereus FORC_024	sulfurtransferase	1,618,123..1,618,305	1,618,327..1,618,510	glutathionylspermidine synthase
Bacillus cereus TG1-6	sulfurtransferase	698,980..699,162 (complement)	698,775..698,958 (complement)	glutathionylspermidine synthase
Bacillus cereus HN001	sulfurtransferase	1,506,719..1,506,901	1,506,923..1,507,106	glutathionylspermidine synthase
Bacillus cytotoxicus CH_13	sulfurtransferase	1,411,733..1,411,916	1,411,937..1,412,120	glutathionylspermidine synthase
Bacillus cytotoxicus CVUAS 2833	sulfurtransferase	608,007..608,190	608,211..608,394	glutathionylspermidine synthase
Bacillus mycoides AH621	sulfurtransferase	1,481,034..1,481,216	1,480,829..1,481,012	glutathionylspermidine synthase
Bacillus mycoides WSBC10204	sulfurtransferase	3,254,178..3,254,360	3,253,973..3,254,156	glutathionylspermidine synthase
Bacillus pseudomycoides DSM 12442	sulfurtransferase	1,465,374..1,465,557	1,465,578..1,465,761	glutathionylspermidine synthase
Bacillus pseudomycoides Rock3-17	sulfurtransferase	1,362,869..1,363,051	1,362,664..1,362,847	glutathionylspermidine synthase
Bacillus wiedmannii SR52	sulfurtransferase	1,522,305..1,522,487	1,522,509..1,522,692	glutathionylspermidine synthase
Bacillus wiedmannii MM3	sulfurtransferase	1,427,163..1,427,345 (complement)	1,426,958..1,427,141 (complement)	glutathionylspermidine synthase

Table S5. Gene organization of *ssrSA* and *ssrSB* in *B. subtilis* group bacteria.

Strains	Expression product of the upstream gene for *ssrSA*	The Location of *ssrSA* on the genome	Expression product of the downstream gene for *ssrSA*	Expression product of the upstream gene for *ssrSB*	The Location of *ssrSB* on the genome	Expression product of the downstream gene for *ssrSB*
Bacillus amyloliquefaciens DSM 7	FMN-dependent NADH-azoreductase	2,096,990..2,097,175 (complement)	DNA helicase RecQ	aspartate-tRNA ligase	2,609,464..2,609,656 (complement)	tRNA threonylcarbamoyladenosine dehydratase
Bacillus amyloliquefaciens WF02	FMN-dependent NADH-azoreductase	2,051,902..2,052,087 (complement)	DNA helicase RecQ	aspartate-tRNA ligase	2,660,582..2,660,774 (complement)	tRNA threonylcarbamoyladenosine dehydratase
Bacillus amyloliquefaciens FS1092	FMN-dependent NADH-azoreductase	4,018,519..4,018,704	DNA helicase RecQ	aspartate-tRNA ligase	3,271,649..3,271,840	tRNA threonylcarbamoyladenosine dehydratase
Bacillus licheniformis DSM 13	multidrug resistance efflux transporter family protein	1,929,675..1,929,858	TetR/AcrR family transcriptional regulator	aspartate-tRNA ligase	2,770,080..2,770,276 (complement)	tRNA threonylcarbamoyladenosine dehydratase
Bacillus licheniformis SRCM100141	multidrug resistance efflux transporter family protein	1,245,984..1,246,167	TetR/AcrR family transcriptional regulator	aspartate-tRNA ligase	2,122,684..2,122,880 (complement)	tRNA threonylcarbamoyladenosine dehydratase
Bacillus licheniformis SCDB 34	multidrug resistance efflux transporter family protein	307,462..307,645	TetR/AcrR family transcriptional regulator	aspartate-tRNA ligase	1,365,476..1,365,672 (complement)	tRNA threonylcarbamoyladenosine dehydratase
Bacillus pumilus SH-B9	TraR/DksA C4-type zinc finger protein	1,935,351..1,935,536 (complement)	DNA helicase RecQ	aspartate-tRNA ligase	2,411,970..2,412,150 (complement)	tRNA threonylcarbamoyladenosine dehydratase
Bacillus pumilus NCTC10337	TraR/DksA C4-type zinc finger protein	1,915,730..1,915,915 (complement)	DNA helicase RecQ	aspartate-tRNA ligase	2,438,604..2,438,784 (complement)	tRNA threonylcarbamoyladenosine dehydratase
Bacillus pumilus SH-B11	TraR/DksA C4-type zinc finger protein	1,987,341..1,987,526 (complement)	DNA helicase RecQ	aspartate-tRNA ligase	2,526,320..2,526,500 (complement)	tRNA threonylcarbamoyladenosine dehydratase
Bacillus subtilis 168	FMN-dependent NADH-azoreductase	2,095,909..2,096,111 (complement)	DNA helicase RecQ	aspartate-tRNA ligase	2,814,491..2,814,691 (complement)	tRNA threonylcarbamoyladenosine dehydratase

Bacillus subtilis NCIB 3610	FMN-dependent NADH-azoreductase	2,095,959..2,096,145 (complement)	DNA helicase RecQ	aspartate-tRNA ligase	2,814,508..2,814,700 (complement)	tRNA threonylcarbamoyladenosine dehydratase
Bacillus subtilis H1	FMN-dependent NADH-azoreductase	362,626..362,812 (complement)	DNA helicase RecQ	aspartate-tRNA ligase	976,207..976,399 (complement)	tRNA threonylcarbamoyladenosine dehydratase
Bacillus subtilis CW14	FMN-dependent NADH-azoreductase	355,291..355,477	DNA helicase RecQ	aspartate-tRNA ligase	4,025,666..4,025,858	tRNA threonylcarbamoyladenosine dehydratase
Bacillus subtilis SRCM102754	FMN-dependent NADH-azoreductase	880,161..880,347	DNA helicase RecQ	aspartate-tRNA ligase	126,047..126,239	tRNA threonylcarbamoyladenosine dehydratase
Bacillus subtilis MB9_B1	FMN-dependent NADH-azoreductase	2,123,635..2,123,821 (complement)	DNA helicase RecQ	aspartate-tRNA ligase	2,859,797..2,859,989 (complement)	tRNA threonylcarbamoyladenosine dehydratase
Bacillus subtilis BS49	FMN-dependent NADH-azoreductase	2,113,939..2,114,125 (complement)	DNA helicase RecQ	aspartate-tRNA ligase	2,832,499..2,832,691 (complement)	tRNA threonylcarbamoyladenosine dehydratase
Bacillus subtilis KKD1	FMN-dependent NADH-azoreductase	2,153,037..2,153,223 (complement)	DNA helicase RecQ	aspartate-tRNA ligase	2,815,969..2,816,161 (complement)	tRNA threonylcarbamoyladenosine dehydratase
Bacillus subtilis BL-01	FMN-dependent NADH-azoreductase	1,522,359..1,522,545	DNA helicase RecQ	aspartate-tRNA ligase	964,894..965,086	tRNA threonylcarbamoyladenosine dehydratase
Bacillus subtilis 2014-3557	FMN-dependent NADH-azoreductase	2,076,299..2,076,485 (complement)	DNA helicase RecQ	aspartate-tRNA ligase	2,839,482..2,839,674 (complement)	tRNA threonylcarbamoyladenosine dehydratase
Bacillus velezensis CBMB205	FMN-dependent NADH-azoreductase	1,898,599..1,898,784	DNA helicase RecQ	aspartate-tRNA ligase	1,327,225..1,327,416	tRNA threonylcarbamoyladenosine dehydratase
Bacillus velezensis CBMB205	FMN-dependent NADH-azoreductase	1,898,599..1,898,784	DNA helicase RecQ	aspartate-tRNA ligase	1,327,225..1,327,416	tRNA threonylcarbamoyladenosine dehydratase
Bacillus velezensis 10075	FMN-dependent NADH-azoreductase	1,179,224..1,179,409	DNA helicase RecQ	aspartate-tRNA ligase	577,077..577,268	tRNA threonylcarbamoyladenosine dehydratase
Bacillus velezensis CGMCC 11640	FMN-dependent NADH-azoreductase	2,179,613..2,179,798 (complement)	DNA helicase RecQ	aspartate-tRNA ligase	2,886,587..2,886,778 (complement)	tRNA threonylcarbamoyladenosine dehydratase
Bacillus velezensis DSYZ	FMN-dependent NADH-azoreductase	2,179,331..2,179,516 (complement)	DNA helicase RecQ	aspartate-tRNA ligase	2,861,667..2,861,858 (complement)	tRNA threonylcarbamoyladenosine dehydratase
Bacillus	FMN-dependent	2,207,686..2,207	DNA	aspartate-tR	2,805,337..2,805	tRNA

velezensis<br>Lzh-a42	NADH-azoredu<br>ctase	,871<br>(complement)	helicase<br>RecQ	NA ligase	,528<br>(complement)	threonylcarbamoylade<br>nosine dehydratase
Bacillus<br>*velezensis*<br>W1	FMN-dependent<br>NADH-azoredu<br>ctase	2,226,035..2,226<br>,220<br>(complement)	DNA<br>helicase<br>RecQ	aspartate-tR<br>NA ligase	2,864,012..2,864<br>,203<br>(complement)	tRNA<br>threonylcarbamoylade<br>nosine dehydratase
Bacillus<br>*velezensis*<br>SRCM103616	FMN-dependent<br>NADH-azoredu<br>ctase	2,212,548..2,212<br>,733<br>(complement)	DNA<br>helicase<br>RecQ	aspartate-tR<br>NA ligase	2,809,214..2,809<br>,405<br>(complement)	tRNA<br>threonylcarbamoylade<br>nosine dehydratase

Table S6. Gene organization of *ssrSA* and *ssrSB* in other bacterial species of *Bacillus* genus.

Strains	Expression product of the upstream gene for *ssrSA*	The Location of *ssrSA* on the genome	Expression product of the downstream gene for *ssrSA*	Expression product of the upstream gene for *ssrSB*	The Location of *ssrSB* on the genome	Expression product of the downstream gene for *ssrSB*
Bacillus coagulans 36D1	rhodanese-like domain-containing protein	2,576,832..2,577,026	chloride channel protein	aspartate-tRNA ligase	2,922,662..2,922,846	bile acid:sodium symporter family protein
Bacillus methanolicus MGA3	elongation factor P	2,277,838..2,278,026 (complement)	stage III sporulation protein AA	aspartate-tRNA ligase	2,436,873..2,437,061 (complement)	replication-associated recombination protein A
Bacillus vietnamensis 151-6	peptidylprolyl isomerase	2,426,465..2,426,652 (complement)	HAD family hydrolase	aspartate-tRNA ligase	3,045,476..3,045,665 (complement)	tRNA threonylcarbamoyladenosine dehydratase
Bacillus vietnamensis HD-02	peptidylprolyl isomerase	1,402,895..1,403,082	HAD family hydrolase	aspartate-tRNA ligase	702,739..702,928	tRNA threonylcarbamoyladenosine dehydratase
Bacillus marisflavi JCM 11544	peptidylprolyl isomerase	884,092..884,279	serine/threonine protein phosphatase	aspartate-tRNA ligase	321,362..321,551	sodium:proton exchanger
Bacillus marisflavi Bac144	FkbM family methyltransferase	3,494,391..3,494,577 (complement)	Fur-regulated basic protein FbpA	aspartate-tRNA ligase	4,406,007..4,406,193 (complement)	tRNA threonylcarbamoyladenosine dehydratase
Bacillus marisflavi 151-25	peptidylprolyl isomerase	2,353,601..2,353,788 (complement)	metallophosphoesterase	aspartate-tRNA ligase	2,974,341..2,974,530 (complement)	Na+/H+ antiporter NhaC family protein
Bacillus marisflavi Marseille-P794	peptidylprolyl isomerase	1,447,551..1,447,738 (complement)	serine/threonine protein phosphatase	aspartate-tRNA ligase	2,010,194..2,010,383 (complement)	Na+/H+ antiporter NhaC family protein
Bacillus marisflavi CH108_3D	peptidylprolyl isomerase	227,706..227,893 (complement)	serine/threonine protein phosphatase	aspartate-tRNA ligase	917,855..918,044 (complement)	Na+/H+ antiporter NhaC family protein
Bacillus weihaiensis Alg07	alanyl-tRNA editing protein	2,788,424..2,788,611 (complement)	bile acid:sodium symporter family protein	aspartate-tRNA ligase	1,235,657..1,235,845	tRNA threonylcarbamoyladenosine dehydratase
Bacillus	MFS	2,732,765..2,732	energy	aspartate-tRN	1,696,242..1,696	tRNA

shackletonii LMG 18435	transporter	,961 (complement)	coupling factor transporter S component ThiW	A ligase	,431	threonylcarbamoyladenosine dehydratase
Bacillus badius DSM 30822	Holliday junction branch migration protein RuvA	1,046,409..1,046,594	50S ribosomal protein L33	aspartate-tRNA ligase	1,022,130..1,022,319 (complement)	tRNA threonylcarbamoyladenosine dehydratase
Bacillus badius SGD-V-25	Holliday junction branch migration protein RuvA	128,648..128,833 (complement)	50S ribosomal protein L33	aspartate-tRNA ligase	152,822..153,011	tRNA threonylcarbamoyladenosine dehydratase

Figure S1

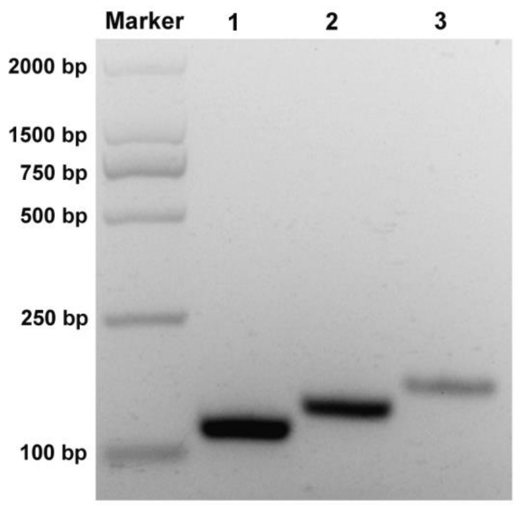

Figure S1. The co-transcription of *ssrSA* and *ssrSB* genes as verified by SqRT-PCR. The TRIzol reagent (life technologies, USA) was used to extract total RNA from BMB171 cells grown in the logarithmic phase, followed by using the PrimeScript RT kit (Takara Biotechnology, Japan) to synthesize cDNAs. The obtained cDNAs were then served as template for PCR amplification using specific primers listed in Table S1, with the results displayed by DNA agarose gel electrophoresis. Lines 1-3 represent *ssrSA*, *ssrSB,* and *ssrSAB*, respectively.

Figure S2

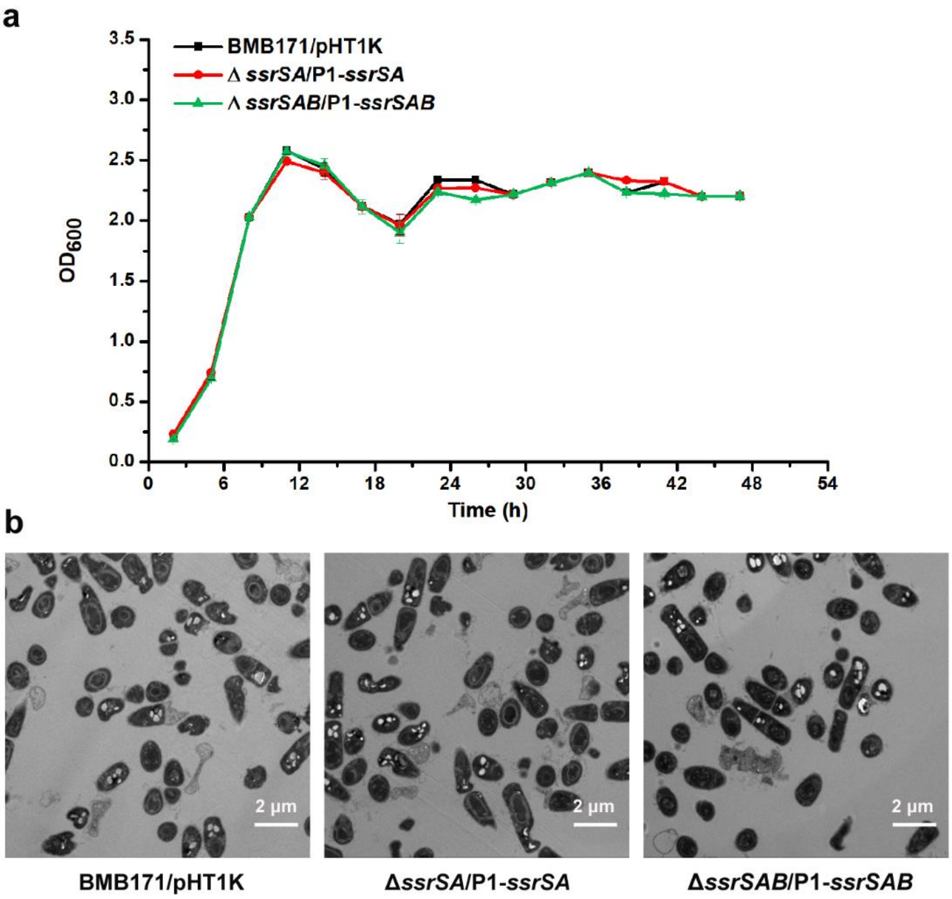

Figure S2. The growth and morphology of complemented strains Δ*ssrSA*/P1-*ssrSA* and Δ*ssrSAB*/P1-*ssrSAB*. **(a)** The growth curves of the complemented strains Δ*ssrSA*/P1-*ssrSA* and Δ*ssrSAB*/P1-*ssrSAB* and the control strain BMB171/pHT1K. The values were means ± standard deviations for triplicate assays. **(b)** The cell morphologies of BMB171/pHT1K, Δ*ssrSA*/P1-*ssrSA,* and Δ*ssrSAB*/P1-*ssrSAB* in the stationary phase (17 h) as observed by transmission electron microscope. The above-mentioned strains were cultured at 28°C in GYS medium.

Figure S3

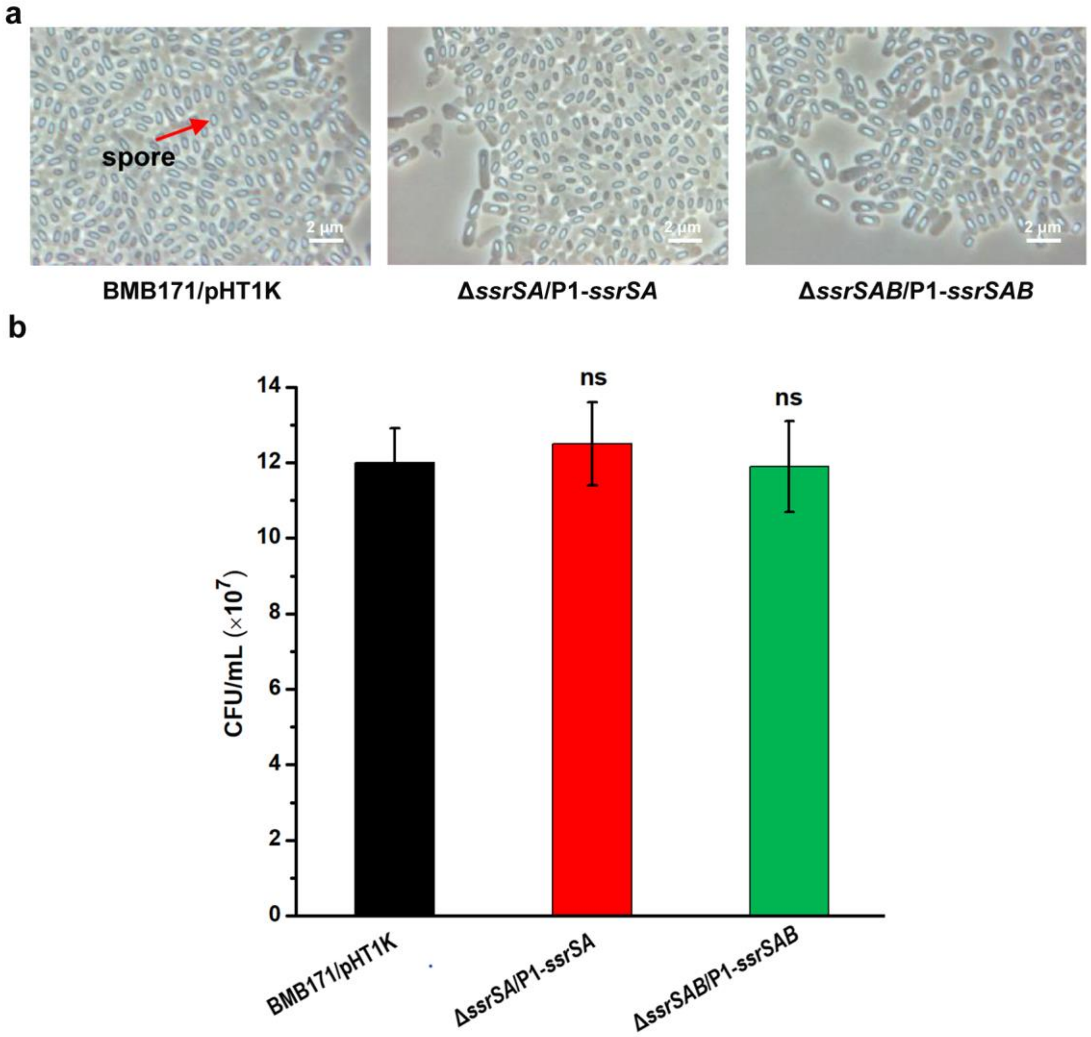

Figure S3. The effect of complementation of *ssrSA* and *ssrSB* genes back to the corresponding deletion mutants on the sporulation of *Bacillus thuringiensis*. **(a)** Observation of the spore formed by the complemented strains, Δ*ssrSA*/P1-*ssrSA* and Δ*ssrSAB*/P1-*ssrSAB* and the control strain BMB171/pHT1K at 24 h (in stationary phase) by phase contrast microscope. Spores were indicated by red arrows. (**b)** The spore counts of strains BMB171/pHT1K, Δ*ssrSA*/P1-*ssrSA,* and Δ*ssrSAB*/P1-*ssrSAB* at 24 h (in stationary phase). The above-mentioned strains were cultured at 28°C in GYS medium. The values were means ± standard deviations for triplicate assays. Significances of differences by Student's t-test were indicated. ***P<0.001; **P<0.01; *P<0.05; ns, P> 0.05.

Figure S4

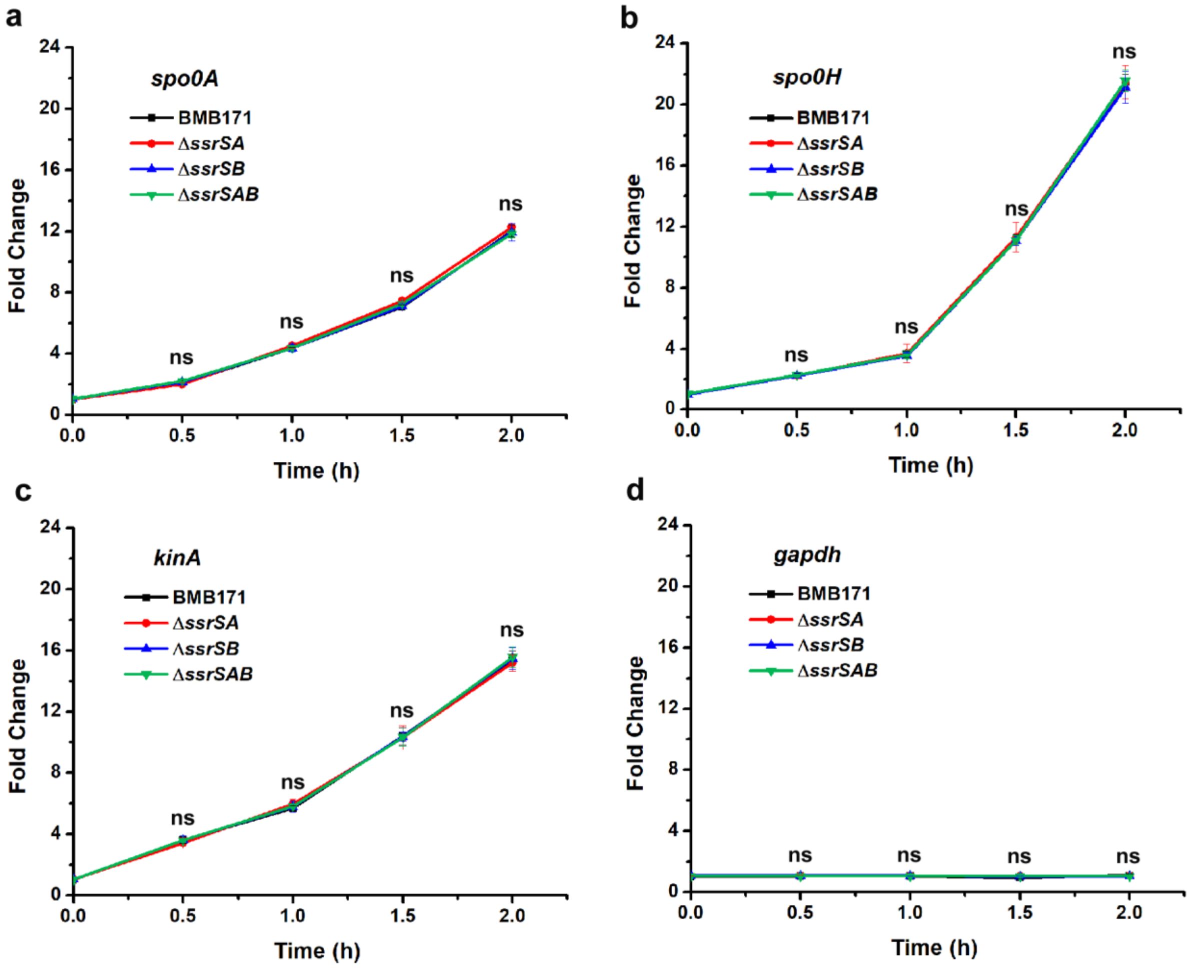

Figure S4. The relative expression levels of several key early sporulation-related genes of BMB171 and its mutant strains Δ*ssrSA*, Δ*ssrSB,* and Δ*ssrSAB* as determined by RT-qPCR. **(a-c)** respectively represent the relative transcription levels of *spo0A* **(a)**, *spo0H* **(b)**, and *kinA* **(c)** in BMB171 and its mutant strains Δ*ssrSA*, Δ*ssrSB* and Δ*ssrSAB* with *gapdh* **(d)** as an internal control. The above-mentioned strains were cultured at 28°C in GYS medium. The time shown is a few hours after the beginning of the transition phase (11 h), and the mRNA level were given as the fold change in multiples relative to the mRNA level of BMB171 at t = 0. The values were means ± standard deviations for triplicate assays. Significances of differences by Student's t-test were indicated. ***P<0.001; **P<0.01; *P< 0.05; ns, P> 0.05.

Figure S5

GAGUAAAUAAUACAUCCUGCGGUGUGCGUAACUUAUUUAUGUCUAAAACCGAUGUUAGUUAUACGGAAGCUCAA

6S-1 RNA

UAUUUAGCGACCAUCAUCAAGCCUUCCUUGUGGAGGAAGAUGUACGGUAACUGUGAGGGCAUCCACCUGCGAGU

AGCGGGUUUUUGGACAUUUACGAGGAGCGGCACGUGCGGGGGUCUUAUUUCAACUAACCUUAUAUAAAAUUCCU

6S-2 RNA

ACGGUGUACGUAGCUUAUGCAUGUCUUAAACCAAUAAGUUUAAUAUGGAAGUUCAAUAUUUAAAUGUAGCUAACA

UGCCAUCUUUUUAAGAAGGAAGUUACAAACAUUUGUGAGAGCAUCCACCUGUGAGAGCAGGUUUAUGGACAUCU

AGAGAGAACGGCAUAUGUGGGGCUAUAUAAAAA

Figure S5. The co-transcript sequence of 6S-1 and 6S-2 RNAs. Both the 5'- and 3'-ends of 6S-1 and 6S-2 precursor RNAs s are rich with A and U nucleotides (were underlined in green). The sequences of mature 6S-1 and 6S-2 RNAs are marked in red and blue, respectively.

Figure S6

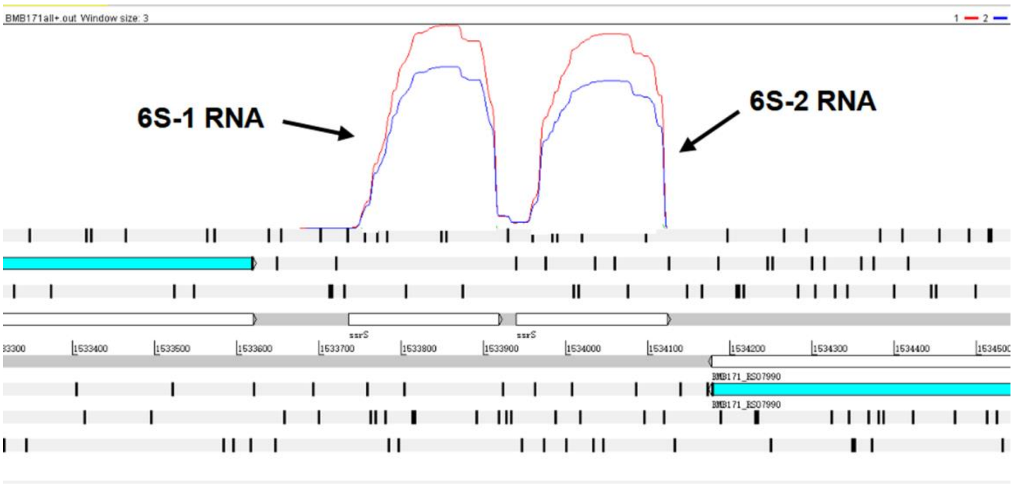

Figure S**6**. Mature 6S-1 and 6S-2 RNAs obtained by strand-specific RNA-Seq. The transcripts are mapped to the *ssrSA* and *ssrSB* genes. The red and blue curves represent the transcription profiles of two duplicate RNA-seq data. RNA-seq data are displayed by Artemis software 17.0 version.

Figure S7

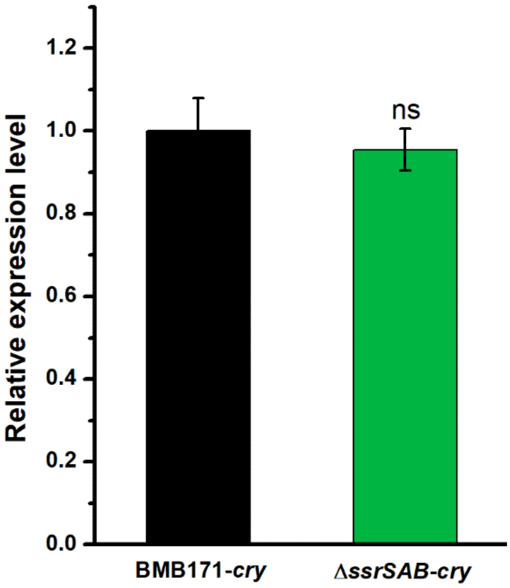

Figure S7. The relative expression levels of *cry1Ac10* in BMB171 and Δ*ssrSAB* as determined by RT-qPCR. The values were means ± standard deviations for triplicate assays. Significances of differences by Student's t-test were indicated. ***P<0.001; **P<0.01; *P< 0.05; ns, P> 0.05.
